# A high quality Arabidopsis transcriptome for accurate transcript-level analysis of alternative splicing

**DOI:** 10.1093/nar/gkx267

**Published:** 2017-04-11

**Authors:** Runxuan Zhang, Cristiane P. G. Calixto, Yamile Marquez, Peter Venhuizen, Nikoleta A. Tzioutziou, Wenbin Guo, Mark Spensley, Juan Carlos Entizne, Dominika Lewandowska, Sara ten Have, Nicolas Frei dit Frey, Heribert Hirt, Allan B. James, Hugh G. Nimmo, Andrea Barta, Maria Kalyna, John W. S. Brown

**Affiliations:** 1 Informatics and Computational Sciences, The James Hutton Institute, Invergowrie, Dundee DD2 5DA, UK; 2 Plant Sciences Division, College of Life Sciences, University of Dundee, Invergowrie, Dundee DD2 5DA, UK; 3 Max F. Perutz Laboratories, Medical University of Vienna, Dr. Bohrgasse 9/3, 1030 Vienna, Austria; 4 The Donnelly Centre, University of Toronto, 160 College Street, Toronto, Ontario, Canada; 5 Cell and Molecular Sciences, The James Hutton Institute, Invergowrie, Dundee DD2 5DA, UK; 6 Centre for Gene Regulation and Expression, School of Life Sciences, University of Dundee, Dundee, UK; 7 Institute of Plant Sciences Paris Saclay, INRA-CNRS-UEVE, Orsay 91405, France; 8 Institute of Molecular, Cell and Systems Biology, College of Medical, Veterinary and Life Sciences, University of Glasgow, Glasgow G12 8QQ, UK; 9 Department of Applied Genetics and Cell Biology, University of Natural Resources and Life Sciences - BOKU, Muthgasse 18, 1190 Vienna, Austria

## Abstract

Alternative splicing generates multiple transcript and protein isoforms from the same gene and thus is important in gene expression regulation. To date, RNA-sequencing (RNA-seq) is the standard method for quantifying changes in alternative splicing on a genome-wide scale. Understanding the current limitations of RNA-seq is crucial for reliable analysis and the lack of high quality, comprehensive transcriptomes for most species, including model organisms such as Arabidopsis, is a major constraint in accurate quantification of transcript isoforms. To address this, we designed a novel pipeline with stringent filters and assembled a comprehensive Reference Transcript Dataset for Arabidopsis (AtRTD2) containing 82,190 non-redundant transcripts from 34 212 genes. Extensive experimental validation showed that AtRTD2 and its modified version, AtRTD2-QUASI, for use in Quantification of Alternatively Spliced Isoforms, outperform other available transcriptomes in RNA-seq analysis. This strategy can be implemented in other species to build a pipeline for transcript-level expression and alternative splicing analyses.

## INTRODUCTION

In plant and animal genomes, the majority of intron-containing genes undergo alternative splicing (AS). AS of precursor messenger RNAs (pre-mRNAs) can generate different transcript isoforms by selection of alternative splice sites (1–3). A major consequence of AS is that mRNA variants are translated to produce different protein isoforms often with different or even antagonistic functions (1–3). In addition to increasing protein complexity, AS can regulate transcript (and consequently protein) abundance by producing transcript isoforms which are degraded by the nonsense-mediated mRNA decay (NMD) pathway (4–7).

In higher plants, the significance of AS as a key regulator of gene expression is illustrated by 60–70% of intron-containing genes undergoing AS (8,9) and 13–18% being regulated by AS coupled to NMD (5,10). AS is important in normal growth and development as well as in responses to biotic and abiotic stresses (11–16). It is involved in, for example, flowering time, the circadian clock, light signalling, seed dormancy, disease resistance and stress responses (17–30). It is therefore essential that gene expression studies in *Arabidopsis thaliana* and other plant species take full account of the diversity of AS transcripts and assess the dynamic changes in expression at the individual transcript level to better understand how plant processes are controlled.

RNA-sequencing (RNA-seq) allows the assessment of differential expression of genes and transcripts through quantification of transcripts across a broad dynamic range. Nevertheless, accurate genome-wide assembly and quantification of transcript isoforms from RNA-seq data remains a substantial challenge (31). Transcriptome assemblies from short read data are usually performed using one of three approaches: reference-guided, *de novo* assembly or a combination of both (32). With an available genome sequence the quantification of transcripts involves mapping of RNA-seq reads to the genomic reference and then construction of transcript isoforms as the first steps. Transcript expression levels are then inferred based on the number of aligned reads. Current analysis tools that identify and quantify transcripts from reads mapped to a genome include TopHat2/Cufflinks (33–36), RSEM (37,38), eXpress (39), Bayesembler (40) and StringTie (41). However, the determination of transcripts from short reads is often inaccurate and generates incorrect, mis-assembled transcripts and misses *bona fide* transcripts that impact the accuracy of transcript quantification (42–44). For example, the assembly functions of two of the best performing programs, Cufflinks and StringTie, generate 35–50% false positives, and quantification based on these transcript annotations often leads to inaccurate results (43). In particular, for genes with multiple isoforms, the accuracy of transcript inference and quantification is poor (43,44). Therefore, the pipelines for RNA-seq analysis need improvement, and assembled transcripts and their quantification require rigorous experimental validation.

Rapid quantification of known transcripts can be achieved using the Sailfish (45), Salmon (46) or kallisto (47), programs which use lightweight algorithms to quantify the abundance of RNA isoforms (45). These programs require well-annotated transcriptomes for accurate quantification of transcript isoforms. Despite the genomic resources in *A. thaliana*, there is limited information on AS transcripts in existing data repositories. Construction of an initial Reference Transcript Dataset (AtRTD) (now referred to as AtRTD1) demonstrated the value of this approach in giving a high correlation of AS using individual transcript abundances from RNA-seq data analysed with Sailfish and Salmon and experimental data from high resolution (HR) RT-PCR (48). Here, we present a comprehensive high-quality reference transcript dataset (AtRTD2) having an increased number of diverse, high confidence Arabidopsis transcripts with over 82k unique transcripts from around 34k genes. The pipeline for construction includes stringent filtering and quality control measures based on our knowledge of plant intron and splicing characteristics, and extensive experimental validation to reduce the number of false transcripts which could perturb quantification. We also provide a modified version, AtRTD2-QUASI (Quantification of Alternatively Spliced Isoforms), for use with Salmon and kallisto to quantify transcripts, and this output can then be used by AS analysis programs such as SUPPA (49).

## MATERIALS AND METHODS

### Plant material for RNA-seq and datasets

Two different extensive RNA-seq datasets were generated for a range of diverse genetic lines and treatments in the *A. thaliana* Col-0 background (Supplementary Table S1). These two combined datasets included 285 RNA-seq runs obtained from 129 libraries. Dataset 1 was from samples of a time-course of adult Arabidopsis plants (5 weeks old) transferred from 20°C to 4°C (unpublished data). Plants were sampled every 3 h for the 24 h period at 20°C directly before transfer to 4°C, and for the first and fourth days following transfer (26 time-points in 78 libraries—a total of 234 total biological/sequencing repeats) (Supplementary Table S1). Dataset 2 consisted of RNA-seq data from 51 libraries generated from various samples (Supplementary Table S1). The genetic lines were over-expression lines and knockout mutants of the serine–arginine-rich (SR) splicing factor genes, At-*RS31* (AT3G61860) and At-*RS2Z33* (AT2G37340); the mutant of the DNA cytosine methyltransferase MET-1 (AT5G49160), *met1-3*; and mutants of three MAP kinase genes (AT3G45640, AT4G01370 and AT2G43790), *mpk3, mpk4* and *mpk6*. The latter were treated with flg22 or mock-treated, and wild type Col-0 controls were included for all of the above. The total number of 100 bp paired-end reads generated in the two datasets of RNA-seq was 4.76 and 3.73 Bn pairs of reads, respectively, such that a total of ca. 8.5 Bn pairs of reads (17 Bn paired-end reads) entered the assembly pipeline.

### Pipeline for generation of AtRTD2

**Figure 1.**
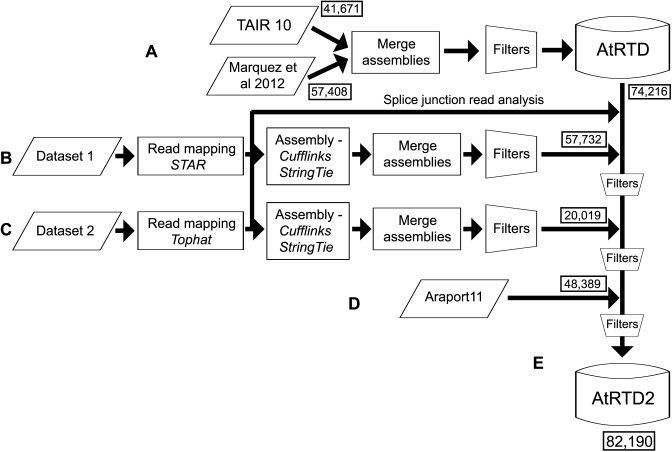
Pipeline for construction of AtRTD2. (**A**) The original AtRTD1 was generated from the merge of transcripts from TAIR10 and from an AS discovery analysis (8) and filtered to remove redundancy (48). Using the splice junction data from the assemblies in (**B**) and (**C**), probable mis-assembled transcripts were removed; (**B**) Dataset 1 RNA-seq reads were mapped with STAR and then assembled using Cufflinks and StringTie. The assemblies were merged and filtered to give a transcriptome; (**C**) Dataset 2 RNA-seq reads were mapped with TopHat2, assembled, merged and filtered as above; the two datasets were analysed separately in different research groups and used different mapping programs (STAR and TopHat2) but both used the transcriptome assembly functions in Cufflinks and StringTie and similar filtering approaches. (**D**) Araport11 transcripts were then merged. Following each transcriptome merge, further filters (e.g. to remove redundancy) were applied (Supplementary Figure S1); (**E**) this generated the final AtRTD2.

A detailed description of the transcript assembly and parameters used, merging with the original AtRTD1 (48) and Araport11 is given in Supplementary Methods and shown schematically in Figure 1. Briefly, RNA-seq reads from Datasets 1 and 2 were mapped to the genome using STAR and TopHat2 respectively, and transcripts for both datasets were assembled with both Cufflinks and StringTie. Transcripts supported by non-canonical junctions or low abundance splice junction reads were removed. Transcripts from unknown genes, antisense transcripts and low abundance transcripts were also filtered out. The resulting Cufflinks and StringTie transcriptome assemblies were merged and redundant transcripts were removed. AtRTD1 was re-assessed using the splice junction sequence set generated here and 10 397 transcripts deriving from Marquez *et al*. (8) were removed. The modified AtRTD1 was then merged with the Dataset 1 transcriptome and then with that of Dataset 2, with a series of quality filters being applied at each step (see Supplementary Methods). Finally, the resulting transcriptome was merged with the Araport11 transcript assembly (50) and filtered again to give the new Arabidopsis transcriptome, AtRTD2.

### Splice site scores from introns in AtRTD2 transcripts

For intron scoring, a total of 3 exonic and 10 intronic nucleotides were extracted from the 5΄ and 3΄ splice site signals for all introns in the AtRTD2 transcripts. The resulting splice site sequences were evaluated using Position Weight Matrices (PWMs) from U2 and U12 canonical signatures (51). A given intron was considered to have a U12 signature only if the 5΄ splice site sequence had a score higher than 75 and the 3΄ splice site sequence had a score higher than 65 in the respective U12 PWM. An intron was considered to have a U2 signature if the scores of the 5΄ and 3΄ splice site sequences were higher than 60 in the corresponding U2 PWM.

### Experimental validation of the quantification of splicing ratios by high resolution RT-PCR

To validate the quantification of splicing ratios from transcript isoforms, HR RT-PCR (52–54) was performed on RNA from two different time-points (each with three biological repeats): dawn at 20°C (T1) and in the middle of the dark period four days after transfer to low temperature (4°C) (T2). T1 and T2 are from the same plant material as used for RNA-seq of Dataset 1. A total of 762 data points (127 AS events from 62 genes and three biological replicates of the two time-points) were analysed by HR RT-PCR using gene-specific primers (Supplementary Table S2). Primer pairs covering the AS events in these genes, where the upstream primer was end-labelled with a fluorescent tag, were used in RT-PCR reactions with 24 cycles of PCR and separated on an ABI 3730 automatic DNA sequencing machine as described previously (5,8,22,52). The abundance of RT-PCR products was analysed with GeneMapper software and splicing ratios were calculated from peak areas of each product. We analyzed the RNA-seq data from the same regions of these 62 genes and used the transcripts per million (TPM) values of the transcripts generated by Salmon on the RNA-seq data to calculate splicing ratios. The splicing ratios were calculated for individual AS transcripts compared to the fully spliced transcript (AS/FS). This was because, in some cases, HR RT-PCR detected relatively low abundance AS transcripts which were not identified in RNA-seq and the AS/FS ratios allowed direct comparisons to be made. Spearman and Pearson correlations were computed on splicing ratios. Manual counting of splice junction reads was performed by visualizing the gene structure and reads using Tablet (55).

### Modification of AtRTD2 for quantification of transcript abundances and generation of AtRTD2-QUASI

The AtRTD2 was modified to examine the effects of transcript 5΄ and/or 3΄ end length variation in genes on isoform quantification. Transcripts were trimmed at the 5΄ end and 3΄ ends. Trimming was to the end co-ordinates of the transcript that covered the shortest region of the gene and was achieved using in-house scripts. Alternatively, transcripts in AtRTD2 were padded to give the transcripts of each gene the same 5΄ and 3΄ ends. Shorter transcripts were extended to the co-ordinates of the transcript that covered the longest region on the gene by adding the cognate genomic sequence by in-house scripts. The AtRTD2-padded version was called AtRTD2-QUASI.

### Translation of AtRTD2 and new peptide database

The main consequences of AS are either to alter the protein-coding sequence to generate protein variants or introduce premature termination codons (PTCs)/long faux 3΄ untranslated regions (UTRs) which can target transcripts to the NMD pathway. In order to assess accurately the consequences of an AS event(s), translation of the transcript from the authentic translation start site is required (56). We therefore developed an algorithm which defined the position of the translation start AUG in the transcripts of a gene and used this translation start site as the reference point for translation of all of the transcripts. The AUG was defined by selecting the transcript which generated the longest open reading frame (ORF). The AUG of this ORF was then used to translate all of the other transcripts of that gene. This method translated ca. 94% of the protein-coding transcripts in AtRTD2. The majority of transcripts which were not translated did not contain the fixed AUG because they were either shorter transcripts or an AS event had removed the AUG. These were then translated by a second part of the programme to identify the longest ORF. The resulting proteins were compared to the current Arabidopsis proteome (57). In addition, the resultant AtRTD2 protein isoforms were converted to a peptide database by *in silico* trypsin digestion using an in-house script. The standard rules of trypsin digestion were applied, in which a given protein is cleaved in every arginine or lysine residue with the exception of those followed by a proline. Only resulting peptides longer than or equal to seven amino acids were kept and mapped back to the original proteins to determine if they can be assigned to a single transcript/protein isoform (unique peptides) or to multiple isoforms.

### Preparation of material for proteomic analysis

Seeds of *A. thaliana* Col-0, an over-expression line of the SR protein, At-RS31 and the *at-rs31* mutant (SALK_021332) (25) were sterilized with 0.6% final concentration of sodium hypochlorite for 5 min, followed by multiple washes with distilled sterile water. Seedlings were grown in the liquid culture with Gamborg's medium, consisting of 3.2 g/l Gamborg's B5 salts with minimal organics, 1 ml/l 1000 × Gamborg's vitamins, 0.5 g/l morpholinoethanesulfonic acid sodium salt, 3% sucrose, pH 5.9 and supplemented with 160 μg/ml l-lysine and 160 μg/ml l-arginine. The Arabidopsis culture was maintained at 22°C, 16 h light/8 h dark cycle and with vigorous shaking, for 19 days (from seeds). Medium was exchanged frequently (every second day after the seeds had germinated). Seedlings of the three lines were harvested and frozen in the liquid nitrogen. Total protein extracts were prepared from 1 g of ground tissue extracted with 1.5 ml of extraction buffer (50 mM Tris–HCl pH 7.6, 0.33 M sucrose, 1 mM MgCl_2_, 1 mM dithiothreitol (DTT), 1% (w/v) C7BzO (Sigma), cOmplete protease inhibitor cocktail (Roche) for 45 min on ice. The lysates were then centrifuged for 10 min at 4000 × g at 4°C, supernatants collected and centrifuged again for 10 min at 18 000 × g at 4°C. A bicinchoninic acid assay (BCA) was performed on the supernatants for determining protein concentration. Equal amounts of proteins extracted from the biological repeats were subjected to SDS-PAGE analysis on 4–12% (w/v) Bis–Tris NuPage gels using 4-morpholinepropanesulfonic acid (MOPS) running buffer (Invitrogen) according to manufacturer's instructions, in the LDS NuPage sample buffer. A maximum of 20 μg of protein was loaded per lane. InstantBlue staining was performed according to manufacturer's instructions (Expedeon). Each lane from the gel was cut into 15 fractions. Gel pieces were de-stained and proteins were reduced with 10 mM DTT and alkylated with 55 mM iodoacetamide. Each gel slice was subjected to in-gel triple digestion with trypsin. The resulted peptides were extracted from gel pieces and cleaned up with in-house C18 columns.

### LC–MS/MS and MaxQuant analysis

A Dionex Ultimate 3000 nanoHPLC system was used with 2 μg of peptides injected onto an Acclaim PepMap C18 nano-trap column (Dionex). After washing with 2% (v/v) acetonitrile 0.1% (v/v) formic acid peptides were resolved on a 150 mm × 75 μm Acclaim PepMap C18 reverse phase analytical column over a 200 min organic gradient with a flow rate of 300 nl/min. Peptides were ionized by nano-electrospray ionization at 1.2 kV using a fused silica emitter with an internal diameter of 5 μm (New Objective). Tandem mass spectrometry analysis was carried out on a LTQ-Velos Orbitrap mass spectrometer (Thermo Scientific) using data-dependent acquisition, measuring and sequencing the top 15 ions. MS/MS raw files were processed and searched using MaxQuant version 1.5.6.51, searching against AtRTD2 translations and the Uniprot *A. thaliana* database (Proteome ID UP000006548, updated October 2016) including further Arabidopsis protein isoform sequences from UniProtKB (updated October 2016). The variable modifications were set as oxidation of methionine and acetylation of the protein N-terminus; fixed modifications were set to carbamidomethylation of cysteines only. The MS tolerance was set to 7 ppm with the MS/MS tolerance set to 0.5Da. The peptide and protein False Discovery Rate (FDR) were both set to 1% (58).

## RESULTS

### Pipeline for generation of AtRTD2

The main objective of generating AtRTD2 was to provide a reference transcript dataset that was as comprehensive and diverse as possible in terms of AS isoforms and contained the highest quality transcripts for gene expression and, in particular, AS analysis (59). To provide diversity of transcripts, two different extensive datasets of ca. 8.5 billion pairs of reads, obtained from 285 RNA-seq runs of 129 libraries (Materials and Methods and Supplementary Table S1), were assembled into transcripts and merged with our previous AtRTD1 (48) and the recently released Araport11 transcript set (50) (Figure 1). The high transcript quality was achieved by applying stringent criteria and filters to minimize the number of false, mis-assembled and poorly supported transcripts, and transcript fragments.

Two different series of quality control filters were applied; the first at the transcript assembly stage and the second at the transcriptome merge stage (Supplementary Figure S1). Following mapping of reads to the genome with STAR and TopHat2, we identified that both Cufflinks and StringTie generated transcripts with novel splice junctions which were unsupported by reads. Therefore, the resulting transcriptome assemblies were initially filtered on the basis of splice junction quality. To this end, we combined the splice junction reads mapped by STAR and TopHat2 and kept only those with canonical splice sites (GT..AG, GC..AG and AT..AC) which were supported by at least ten unique reads in at least three samples, generating ∼220k unique splice junctions. Filtering of the transcript assemblies, based on these splice junctions, resulted in the removal of 33.5% and 49.0%, and 28.3% and 33.6% of the transcripts from the initial Cufflinks and StringTie assemblies for Datasets 1 and 2, respectively (Supplementary Figure S1A). The high numbers of discarded transcript models, containing at least one intron with non-canonical splice sites or which was poorly supported by splice junction reads, correlate well with previous observations that Cufflinks and StringTie generate 35–50% false positives (43). The prediction of such ‘introns’ by these programs highlights the need to quality control assembled transcripts prior to quantification of transcript isoforms. Following the splice junction quality filter, antisense transcripts that were completely contained within an annotated gene in TAIR10 were removed along with transcripts from unknown genes and transcripts with no or very low expression (Supplementary Figure S1A). The latter were identified by analyzing the RNA-seq read data using Salmon and were removed on the basis that they likely represent mis-assembled transcripts. Only the transcripts which had a TPM (transcripts per million) >1 in at least three samples were kept.

At this stage, transcriptomes of around 50k transcripts were generated by Cufflinks and StringTie from the RNA-seq data of Datasets 1 and 2 (Supplementary Figure S2A and B). In each case, 71–79% of the transcripts were identical between the Cufflinks and StringTie assemblies. To generate the final AtRTD2, these transcriptomes were merged together and with AtRTD1 and Araport11 in a step-wise manner (Figure 1), and a second set of splicing and redundancy filters was applied after each merge (Supplementary Figure S1B, and Supplementary Methods). The stringent filters will result in the loss of some transcripts with alternative transcription start sites or polyadenylation sites and of some *bona fide* transcripts which may be only expressed at very low levels or in a small number of specific cells. However, they have been removed currently for the purpose of generating a robust core dataset for transcript level quantification and AS analyses. The final AtRTD2 contains 82 190 unique transcript models.

To show that the introns in the AtRTD2 transcripts predicted by the splice junction reads were *bona fide* introns, we searched for sequence signatures of plant U2 and U12 introns using PWMs (8,51). The majority of predicted introns (>99.8%) have signatures of typical plant introns with PWM values of >60 at both 5΄ and 3΄ splice sites (74.8% with PWM values >65 at each splice site) (Supplementary Table S3). In addition, we identified 41 putative U12 AT..AC and 589 putative U12 GU..AG introns in 156 and 2152 transcripts, respectively (PWM values of >75 and >65 at 5΄ and 3΄ splice sites, respectively).

### Transcript diversity of AtRTD2

The 82 190 unique transcripts in AtRTD2 are made up of 7518 and 10 460 novel transcripts from Datasets 1 and 2, respectively, 33 673 transcripts from AtRTD1, of which 14 872 were from Marquez *et al*. (8) and 18 801 from TAIR10, and 30 538 transcripts from Araport11 reflecting the extended 5΄ and 3΄ UTR sequences generated in Araport11 compared to TAIR10. AtRTD2 contains transcripts from non-coding (nc) RNA genes such as microRNA (miRNA), spliceosomal small nuclear RNA, small nucleolar RNA, transfer RNA genes etc. The majority of these genes do not undergo AS and will have a single transcript isoform. However, although some miRNA and long ncRNA precursors are alternatively spliced (60–62), our main focus is on alternatively spliced protein-coding genes. For the 27 667 protein-coding genes currently annotated in Araport11, AtRTD2 has a total of 74 197 unique transcript isoforms. Thus, there is an average of 2.68 transcripts per protein-coding gene in AtRTD2 reflecting higher transcript isoform complexity than found previously (Table 1). The increased transcript complexity in AtRTD2 compared to TAIR10 and AtRTD1 is also shown by the increased number of genes with higher numbers of transcripts (Supplementary Figure S3). Thus, AtRTD2 represents a non-redundant transcript dataset highly enriched in AS transcripts.

**Table 1.** Number of *Arabidopsis thaliana* genes and transcripts in different datasets

	Number of genes	Number of transcripts	Average number of transcripts per gene
**All genes**			
TAIR10	33 602	41 671^a^	1.24
Marquez *et al*. (8)	23 905	57 408	2.40
AtRTD1	33 625	74 216^b^	2.21
AtRTD2	34 212	82 190	2.40

**Protein-coding genes**			
Araport11	27 667	48 389	1.75
AtRTD2	27 667	74 194	2.68

Number of transcripts and the average number of transcripts per gene are based on the total number of genes (TAIR10, AtRTD1 and AtRTD2) or the total number of genes detected (8) or on protein-coding genes (Araport11 and AtRTD2).

^a^Contains redundant partial transcripts which differ only by lengths of 5΄ and 3΄ UTRs.

^b^Merged, non-redundant transcripts.

**Figure 2.**
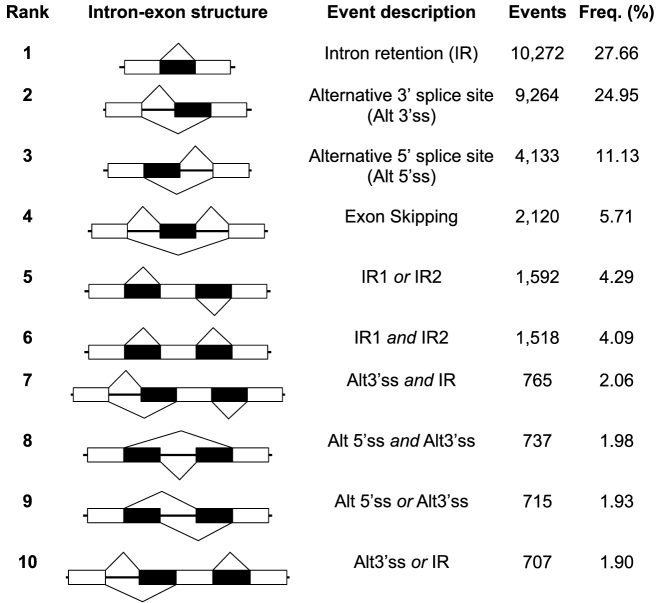
Top 10 most frequent types of AS events/event combinations in AtRTD2 according to AStalavista (63). The intron-exon structure of the AS events are illustrated (exons are denoted by boxes and introns are thick black lines, except AS regions which are shown as black boxes), followed by the event description, the raw number of events found in AtRTD2 and their frequency. Additional AS events are given in Supplementary Table S4.

The transcripts in AtRTD2 contain simple and complex AS events and combinations of AS events. The types of AS events were determined using AStalavista (v4.0.1) (63) to allow a direct comparison to our earlier analysis of the AS landscape in Arabidopsis (8). AtRTD2 contained 37 137 events and those which occurred at least 50 times made up 95.24% of all AS events (Figure 2 and Supplementary Table S4). Intron retention is the most common event (∼40% across the different AS types) followed by alternative 3΄ splice site selection, alternative 5΄ splice site selection and exon skipping agreeing with previous RNA-seq AS analyses (8,64). We also examined the AtRTD2 transcripts for the presence of exitrons in the coding sequences (28) and identified 2459 exitrons making up 6.6% of the AS events identified above.

### Accuracy of quantification of AS with Salmon and AtRTD2

**Figure 3.**
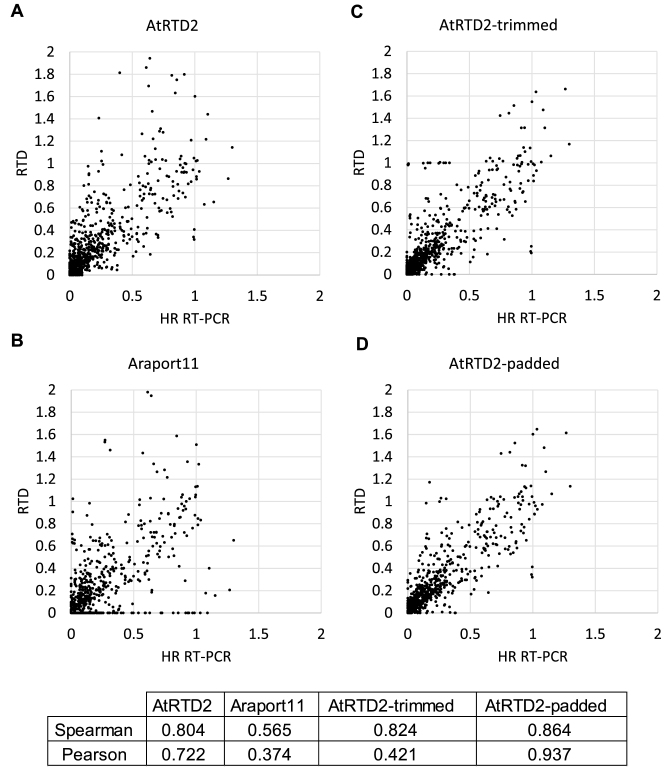
Correlation of splicing ratios calculated from the RNA-seq data and High Resolution Reverse Transcription PCR (HR RT-PCR). Splicing ratios for 127 AS events from 62 *Arabidopsis thaliana* genes (three biological replicates of the time-points T1 and T2) generated 762 data points in total. The splicing ratio of individual AS transcripts to the cognate fully spliced (FS) transcript was calculated from TPMs generated by Salmon and (**A**) AtRTD2, (**B**) Araport11, (**C**) AtRTD2-trimmed and (**D**) AtRTD2-padded and compared to the ratio from HR RT-PCR. Correlation coefficients are given for each plot. Note that for clarity of the figures, 3, 7, 9 and 5 data-points with values that lie substantially outside the range of the graphs are not included in (A)–(D), respectively, but are included in the correlation values. AtRTD2-trimmed and AtRTD2-padded are explained in the text.

To demonstrate the utility of the AtRTD2, we used it to quantify changes in AS transcript ratios with the Salmon quantification tool (46). For experimental validation, we used High Resolution (HR) RT-PCR on the same RNA samples. Previously, HR RT-PCR (52) validated >92% of 586 assembled AS transcripts for 256 genes in Arabidopsis (8). Here, AtRTD2 transcript structures were compared to the amplicons in HR RT-PCR and the TPMs of individual transcripts used to calculate splicing ratios for each of the AS events or event combinations in that region (see Supplementary Figures S4 and S5). To verify the output from Salmon with HR RT-PCR, we analyzed 127 AS events from 62 genes and three biological replicates of the two time-points (T1 and T2, see Materials and Methods), a total of 762 data points. We calculated the splicing ratio for each AS transcript compared to the fully spliced transcript (AS/FS) by comparing the abundance of individual AS transcripts to that of the fully spliced (FS) transcript, which is usually the most abundant transcript and codes for the full-length protein. Correlations values of 0.722 (Pearson's correlation coefficient) and 0.804 (Spearman's rank correlation coefficient) were obtained (Figure 3A and B).

### Missing transcripts impact the accuracy of quantification of AS

**Figure 4.**
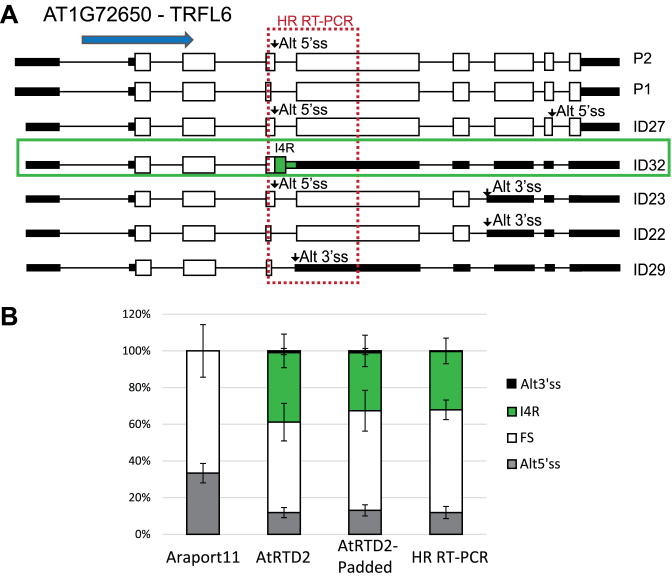
Missing reference transcripts affect transcript and AS quantification. (**A**) AT1G72650 (*TRFL6*) has five different AS events (two Alt5΄ss in exons 4 and 8; two Alt3΄ss in exons 5 and 7, and retention of intron 4 - I4R) contained in seven transcripts. HR RT-PCR across exons 4 and 5 (dashed red box) detects the fully spliced product, Alt5΄ss, Alt3΄ss and I4R. The blue arrow shows the direction of transcription. Where an AS event introduces a PTC disrupting the ORF, the downstream region is represented by black boxes (effectively a UTR). (**B**) AS ratios calculated from TPMs of all seven transcripts from analysis of RNA-seq data (T1 time-point of Dataset 1) using Araport11, AtRTD2 and AtRTD2-padded (explained later in the main text) as reference transcriptomes and compared to HR RT-PCR (T1 time-point). The absence of a transcript with I4R in Araport11 (ID32 in AtRTD2 – green box) affects the quantification of transcripts and AS. Error bars: standard deviation.

To examine whether the quality of AS quantification depends on the completeness and diversity of transcript models in a reference transcriptome, we compared the AS/FS splicing ratios obtained with HR RT-PCR to those derived from TPMs generated by analyzing the same RNA-seq data using Araport11 and AtRTD2 as the reference transcriptomes. We predicted that the smaller number of transcripts in Araport11 (ca. 48.4k transcripts versus ∼82k in AtRTD2) would impact transcript quantification and AS. Indeed, lower correlations of 0.374 (Pearson's) and 0.565 (Spearman's) were obtained with Araport11 (Figure 3A and B). At an individual gene level, the impact of missing transcripts is shown for three genes: *TRFL6*–AT1G72650 (Figure 4), *FRS2* –AT2G32250 and *RVE2* –AT5G37260 (Supplementary Figures S4 and S5). In *TRFL6*, the transcript with retention of intron 4 makes up ∼30% of expressed transcript isoforms but its absence in Araport11 affects the quantification of transcripts and thereby AS (Figure 4). In contrast, in *FRS2*, Araport11 uniquely provides a novel transcript (retention of intron 6) which represents ∼25% of the expressed transcripts from this gene. As expected, analysis of RNA-seq data using AtRTD2 without the Araport11 transcripts shows large changes in the relative abundances of the transcripts and in AS for this gene (Supplementary Figure S4). Finally, in *RVE2*, transcripts with the alternative exon (AT5G37260_ID2, AT5G37260_ID4 and AT5G37260_JC4) are unproductive as the AS event introduces a PTC. The AT5G37260_ID2 transcript makes up ∼80% and 8% of expressed transcript isoforms in T1 and T2 time-points, respectively. Its absence in the TAIR10 and Araport11 transcriptomes results in only the functional protein-coding transcript being reported (Supplementary Figure S5). Therefore, the more comprehensive and complete a reference transcriptome is, the better will be the accuracy of measuring AS isoforms and their contribution to gene expression.

### Variation in UTR length among transcripts affects accuracy of quantification of isoforms and AS events

The correlations obtained with AtRTD2 were unexpectedly lower than those obtained with AtRTD1 (48). We observed that some genes with transcripts with variation in their 5΄ and/or 3΄ UTR lengths showed discrepancies between the splicing ratios obtained from HR RT-PCR and Salmon/AtRTD2. The redundancy filters applied in construction of AtRTD2 gave transcripts with unique intron co-ordinates but which could have different UTR lengths. Such UTR variation may be due to *bona fide* differences in transcript ends or to various artefacts (see Discussion). We made the assumption that, for the majority of genes, much of the variation in 5΄ and 3΄ UTR length of transcripts is likely to be due to transcripts being incomplete and missing terminal regions (i.e. not full-length) and that modifying transcripts so they had the same start and end co-ordinates should improve quantification of isoforms. Therefore, to demonstrate that UTR length variation impacted quantification of such genes, we took two approaches: (i) we trimmed transcripts from the 5΄ and 3΄ ends to the co-ordinates of the transcript(s) that covered the smallest region of the gene (Supplementary Figure S6A and B) or (ii) we padded genomic sequence from the ends of the shorter transcripts up to the co-ordinates of the end of the transcript(s) that covered the biggest region of the gene (Supplementary Figure S7A and B). These modifications were performed transcriptome-wide on AtRTD2 to generate AtRTD2-trimmed and AtRTD2-padded versions, and the RNA-seq data were re-analysed with Salmon and the modified AtRTD2 datasets. Splicing ratios were again calculated and compared to HR RT-PCR for the 127 AS transcripts from 62 genes.

The trimmed version of AtRTD2 improved the Spearman's rank correlation (0.824) compared to AtRTD2 (Figure 3). However, the Pearson's correlation was greatly reduced (0.421). Although trimming of 5΄ and 3΄ ends for many genes resulted in a higher correlation with HR RT-PCR, correlations for some genes were markedly different. By examining the effects of trimming in detail for these genes, we found that trimming could give rise to new UTR sequence length variation affecting quantification (Supplementary Figure S6C and D). For example, when the 5΄ end of a shorter transcript corresponded to a position in a 5΄ UTR intron (Supplementary Figure S6C), trimming to this position often resulted in the longer transcripts losing exon sequences and becoming shorter, thereby introducing new UTR variation (Supplementary Figure S6D). The impact on isoform quantification is illustrated for *XBAT35*–AT3G23280 where trimming to the length of the shortest transcript (AT3G23280.s1) removes exons 1 and 2 of the other transcripts, generating new UTR variation and causing large changes in the TPM values of the transcripts (Supplementary Figure S8).

### Improved AS quantification with AtRTD2-QUASI (Quantification of Alternatively Spliced Isoforms)

**Figure 5.**
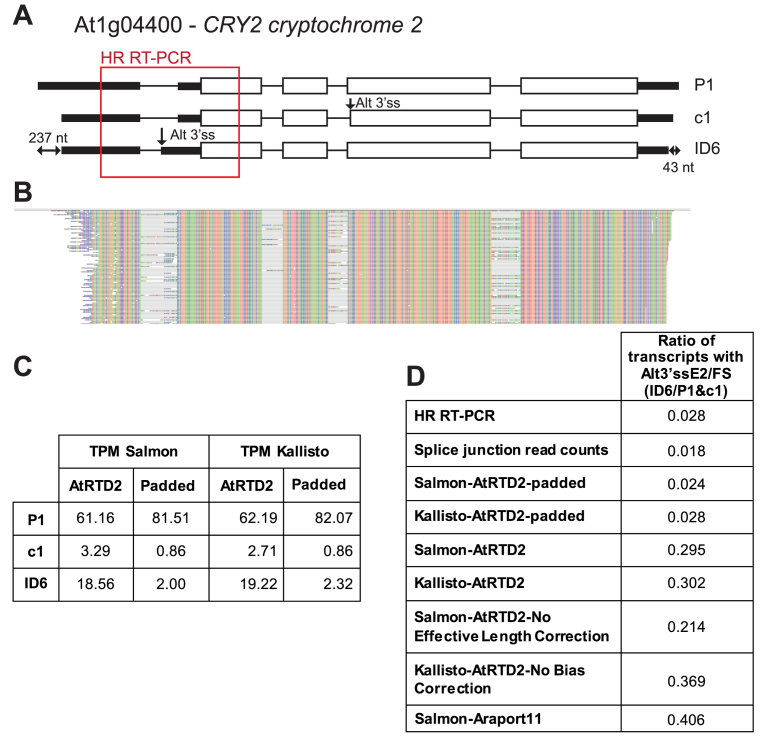
Variation in UTR lengths affects transcript and AS quantification. (**A**) AT1G04400 (*CRY2; CRYPTOCHROME 2*) has two different AS events (an Alt3΄ss in exon 2 and Alt3΄ss in exon 4). The difference between the shortest (ID6) and longest (P1) transcripts is 237 and 43 nt at the 5΄ UTR and 3΄ UTR, respectively. (**B**) Read alignment showing the relatively low level of Alt3΄ss splice junction reads. (**C**) Average TPMs of the 3 transcripts using Salmon and kallisto and the AtRTD2 and AtRTD2-padded; (**D**) AS ratios of the transcript with Alt3΄ss in exon 2 to FS (fully spliced) transcripts with HR RT-PCR, manual counting of splice junction reads in this region using the Tablet read alignment viewer, Salmon and kallisto with AtRTD2-padded and AtRTD2, Salmon and kallisto with uncorrected functions with AtRTD2, and Salmon with Araport11.

We found an overall increased correlation of AS/FS ratios from HR RT-PCR with the AtRTD2-padded version - Spearman's rank correlation and Pearson's correlation coefficients 0.864 and 0.937, respectively (Figure 3). Very similar results were obtained when the RNA-seq data was analysed with kallisto (Supplementary Figure S9). The improved quantification was examined in detail for specific genes/transcripts with different degrees of UTR length variation. TPM values were similar using AtRTD2 and AtRTD2-padded for genes where transcripts had little or no differences in their 5΄ or 3΄ ends (for example see AT5G05550 (*VFP5*) - Supplementary Figure S10). For genes with 5΄ and 3΄ end variation in the first and/or last exon, AtRTD2-padded gave more accurate TPM values. This was clearly shown by the three-way corroboration of AS/FS values from HR RT-PCR, from TPMs from analysing the RNA-seq data with Salmon and kallisto using AtRTD2-padded, and from manual counting of splice junction reads in a read alignment viewer, Tablet (55). For example, there is a 10-fold difference in the AS/FS splicing ratios of the Alt3΄ss event in *CRY2* between the HR RT-PCR data, Salmon/AtRTD2-padded and read counts when compared to analysis with AtRTD2 or Araport11 (Figure 5). Further examples are shown for genes encoding a RING/U-box superfamily protein and *HSF3* (Supplementary Figures S11 and S12). While trimming involving an intron (e.g. in the 5΄ UTR) had negative effects on accuracy of transcript quantification (Supplementary Figures S6C, D and S8), padding of a shorter transcript which ended within an intron did not affect quantification greatly. This was likely due to a transcript ending within a 5΄ or 3΄ UTR intron being indicative of an intron retention event and padding effectively generates the full intron retention (Supplementary Figure S7C, D). For example, AT4G35800 has an intron in the 3΄ UTR (intron 13) and a transcript which terminates in the intron. Padding generates a transcript with retention of intron 13 which has been shown to occur and to be up-regulated in the cold (65). We also examined UTR variation in the genes/transcripts used in HR RT-PCR and observed greatly improved correlation for transcripts where padding has added between 100 and 600 nt at the 5΄ and 3΄ ends (Supplementary Figure S13). We therefore suggest that AtRTD2-padded is a useful tool in quantification of AS transcript isoforms for most genes and release it here as AtRTD2-QUASI specifically for use in Quantification of Alternatively Spliced Isoforms. AtRTD2-QUASI overcomes problems of local variation and heterogeneity in the 5΄ and 3΄ ends of transcripts and improves the accuracy of isoform quantification and thereby differential expression analyses.

Why artificially extended shorter transcripts deliver this increased accuracy is unclear. Most transcript quantification and differential expression programs have algorithms to correct for read distribution variation based on the effective length of transcripts and for bias of read distribution towards the ends of transcripts. Neither of these factors affected the improved accuracy obtained with AtRTD2-QUASI (Figure 5 and Supplementary Figures S11 and S12). This suggests that the phenomenon that we have observed has a different basis or may reflect the robustness of bias models when dealing with genes with multiple isoforms (35,66). Three features may be responsible for the less accurate quantification of transcripts with 5΄ and/or 3΄ UTR variation: longer transcripts containing unique sequences, the different degrees of overlap among multiple transcripts or the inconsistency between read coverage from the experimental data and the longer transcripts from the AtRTD2 which may reflect assembly processes (Supplementary Figure S14).

### Proteomic analysis using AtRTD2 translations

AtRTD2 contains a larger and more diverse set of supported transcripts than has hitherto been available. To investigate the performance of AtRTD2 in proteomic analyses, we firstly characterized the true coding capacity of the AtRTD2 transcripts by translating transcripts from the same gene using the same translation start site AUG. This contrasts the approaches used in TAIR and Araport, where translation programs usually identify the longest ORF in a transcript often leading to erroneous interpretation of transcripts by disregarding PTCs (56). We developed an algorithm which fixed the translation start site AUG for each gene (see Methods). From the ca. 74.1k protein-coding transcripts in AtRTD2, 72.7k (98.1%) were translated into polypeptides (Supplementary Table S5). Around 6.7k transcripts from AtRTD2 were from non-coding RNAs or retrotransposons and were not translated. Secondly, we performed *in silico* trypsin digestion in the 72 724 putative protein isoforms (from 27 434 different genes) from AtRTD2 to generate predicted peptides ≥7 amino acids. This resulted in 569 998 different peptides (Supplementary Table S6), of which 276 232 (48.5%) predicted peptides were associated with a single transcript and represented translations of 38 034 different transcripts from 24 601 genes.

We next performed proteomic analysis of 16-day old seedlings of Col-0, and over-expression and mutant lines of the SR protein, At-RS31 (AT3G61860) (25) grown in liquid culture. The MS/MS spectra were analysed using the translated protein sequences from AtRTD2 and the UniProt Arabidopsis proteome (Proteome ID UP000006548; see Materials and Methods) which contained 27 060 protein sequences. These sequences were supplemented with 2055 Arabidopsis protein isoform sequences from UniProtKB and we refer to the combined UniProt reference as UniProt+. A total of 8693 and 8749 peptides were identified using AtRTD2 and UniProt+, respectively. Over 96% of the peptides ≥7 amino acids (not including peptides with missed cleavages) were identified by both protein isoform sets, with only 115 peptides unique to AtRTD2 and only 171 peptides unique to UniProt+. Thus, the vast majority of peptides are detected by both AtRTD2 and UniProt+. Comparing the two datasets, AtRTD2 contained 57 227 non-redundant protein isoforms while UniProt+ contained 32 539. Similarly, AtRTD2 had 8056 genes with unique peptide support for at least two isoforms while UniProt+ had 2927 such genes. Therefore, although AtRTD2 has greater protein diversity, this is not reflected in the proteomic analysis and probably reflects the detection of proteins from only 2732 genes. We expect that the increased diversity of proteins from AtRTD2 will facilitate isoform detection as the sensitivity of proteomic technologies improves.

## DISCUSSION

In this paper, we report the transcriptome assembly pipeline for generating high quality non-redundant transcriptomes for improved accuracy of RNA-seq analyses. Clearly the quality of transcriptome annotation can have a major impact on expression and AS analysis using RNA-seq (67) and accurate, comprehensive reference transcriptomes are not available for the majority of species which severely limits the exploitation of RNA-seq in expression analysis. The pipeline presented here reduces the number of poorly supported, mis-assembled transcripts and transcript fragments often arising from the high degree of inaccuracy of transcript assembly programs (42,43). The new AtRTD2 contains over 82k non-redundant transcript isoforms from 34 212 genes such that 60% of Arabidopsis protein-coding, intron-containing genes have AS transcripts. Despite Arabidopsis being arguably the best-studied plant species with the most advanced genome and transcriptome annotation, AtRTD2 contains a significantly higher number of transcript isoforms than other collections such as TAIR10 and Araport11. Importantly, we demonstrate the significance of an extensive reference transcript dataset by showing experimentally the drastic effects of missing transcripts on quantification at the individual transcript and gene levels, and at reference transcriptome levels. Although AtRTD2 contains over 82k unique transcripts, it is unlikely to be complete because despite using a range of different sources of transcripts, all possible developmental stages and environmental conditions are not yet covered. Similarly, although deep RNA-seq data and multiple quality control filters have been employed in the pipeline, there are still likely to be mis-assembled and missed transcripts due to the issues with assembly of short read data (42,43). New releases of AtRTD will be generated as other high quality RNA-seq and single molecule sequencing data becomes available.

In assessing the quality of AtRTD2 in analysing RNA-seq data, extensive validation of transcript isoforms and their abundance has been critical. Previously, we developed HR RT-PCR for analysis of changes in AS (5,22,25,52,68,69) and have used it to validate transcript assemblies from RNA-seq data (8,48). Here, we showed generally good correlation of splicing ratios calculated from TPMs from RNA-seq data with Salmon/AtRTD2 and the HR RT-PCR. However, this detailed analysis identified discrepancies in the accuracy of quantification of transcripts from the same gene having different 5΄ and/or 3΄ UTRs and that correction functions within quantification programmes do not appear to deal effectively with this problem. Thus, current RNA-seq analyses in Arabidopsis are likely to be inaccurate and we anticipate that this problem will affect the accuracy of RNA-seq analyses in other species with incomplete and poorly annotated transcriptomes. An example of the effects of transcript length variation (edge bias) on transcript quantification with RNA-seq have been observed for some human transcripts in quantification of AS due to the disproportionate assignment of reads to transcripts with and without UTR sequences (49). Here, we observe that longer 5΄ or 3΄ UTRs are often associated with few or no reads such that these transcripts may be seen as low abundance transcripts, affecting the accuracy of isoform quantification.

Variation in UTR sequences among transcripts from the same gene can occur in many ways. Firstly, some will reflect the use of *bona fide* alternative transcription start sites and alternative polyadenylation. Methods such as 5΄ RACE have demonstrated alternative start sites for a small number of genes but genome-wide information is not extensive (70). Transcription can also be stochastic, often starting in a region of the promoter with no one single nucleotide being the transcription start site such that transcripts may already have variation in the 5΄ UTR (70). Variation in the 3΄ UTR can be generated by alternative polyadenylation. In Arabidopsis, genome-wide information is available from direct RNA sequencing and ca. 75% of genes have more than one polyadenylation site but most reads were associated with a preferred site (71). Indeed, variation in polyA sites is often associated with multiple overlapping polyA signals perhaps ensuring termination in the compact genome (71). Legacy transcripts, such as those in TAIR10, are derived from cDNA/EST cloning and sequencing and some contain no annotated UTRs, although much of this latter variation has been re-annotated (71) and incorporated in the Araport11 transcripts. Secondly, UTR variation may also arise from artefacts of reverse transcription/internal priming, different protocols of cDNA library preparation prior to RNA-seq and *in vivo* or *in vitro* RNA degradation. Finally, variation in the UTRs may arise from mis-assembly or mis-annotation of UTRs during transcript assembly.

To overcome the problem of accurate quantification of transcripts with UTR variation, we tested and validated AtRTD2 modifications iteratively and developed AtRTD2-QUASI that greatly improves the accuracy of AS quantification. We propose that RNA-seq data from Arabidopsis is analysed with Salmon or kallisto using AtRTD2-QUASI because transcript level quantification data agrees well with data obtained experimentally (HR RT-PCR) and data derived from manually counting splice junction reads. The three-way corroboration of results suggests that AtRTD2-QUASI is a practical solution to obtaining good quantitative data on AS transcripts and this approach can be applied to other species. Although AtRTD2-QUASI improves the quantification markedly for the majority of genes, because of the assumption on which it is based, it may not be appropriate for quantification of transcripts with *bona fide* alternative transcription start sites or different polyadenylation sites.

Finally, we have extended the utility of AtRTD2 by generating realistic translations from the AS transcripts in AtRTD2. Fixing the translation start site in transcripts from the same gene generates translations which more accurately reflect the transcript structures and ORFs in different AS isoforms (e.g. AS events that are in frame, change frame or generate PTCs). This approach overcomes problems of mis-interpretation of transcripts due to mis-annotation of ORFs (56). In addition, proteomic analyses using these translations of the AtRTD2 transcriptome and UniProt+ identified effectively the same set of proteins and we expect that as the sensitivity of proteomic technologies improves, the greater diversity of AtRTD2 will benefit proteomic analyses. Taken together, we have demonstrated the impact that incomplete transcriptomes can have on downstream analyses and that, therefore, transcriptomes need to be of the highest quality and constantly refined with new data. Our general strategy and pipeline of construction and experimental validation of a high quality reference transcriptome for expression and AS analyses using RNA-seq data (59) could be important in many other species.

## AVAILABILITY

The Reference Transcript Datasets AtRTD2 and AtRTD2-QUASI are available in the James Hutton Institute repository, [http://ics.hutton.ac.uk/atRTD/]. The translation script fixing translation start sites is available at https://github.com/Dundee-alt-splicing/AtRTD2_translation_pipeline.

## ACCESSION NUMBERS

Raw RNA-seq data files have been uploaded to European Nucleotide Archive (ENA) under the accession number of PRJEB19974 (Dataset 1) and to the Short Read Archive under the accession numbers PRJNA379206, PRJNA379224 and PRJNA379910 for the SR, MET1, MKK/flg22 material, respectively (Dataset 2).

## Supplementary Material

Supplementary Data
