# Supplementary material for: A high quality Arabidopsis transcriptome for accurate transcript-level analysis of alternative splicing
Source: Nucleic Acids Res. 2017 Apr 11;45(9):5061–73. doi: 10.1093/nar/gkx267 (PMC5435985; doi:10.1093/nar/gkx267)
Supplement: Supplementary Data [file gkx267_Supp.zip › nar-03123-z-2016-File007.pdf]

A high quality Arabidopsis transcriptome for accurate transcript-level analysis of alternative splicing

Supplementary Data

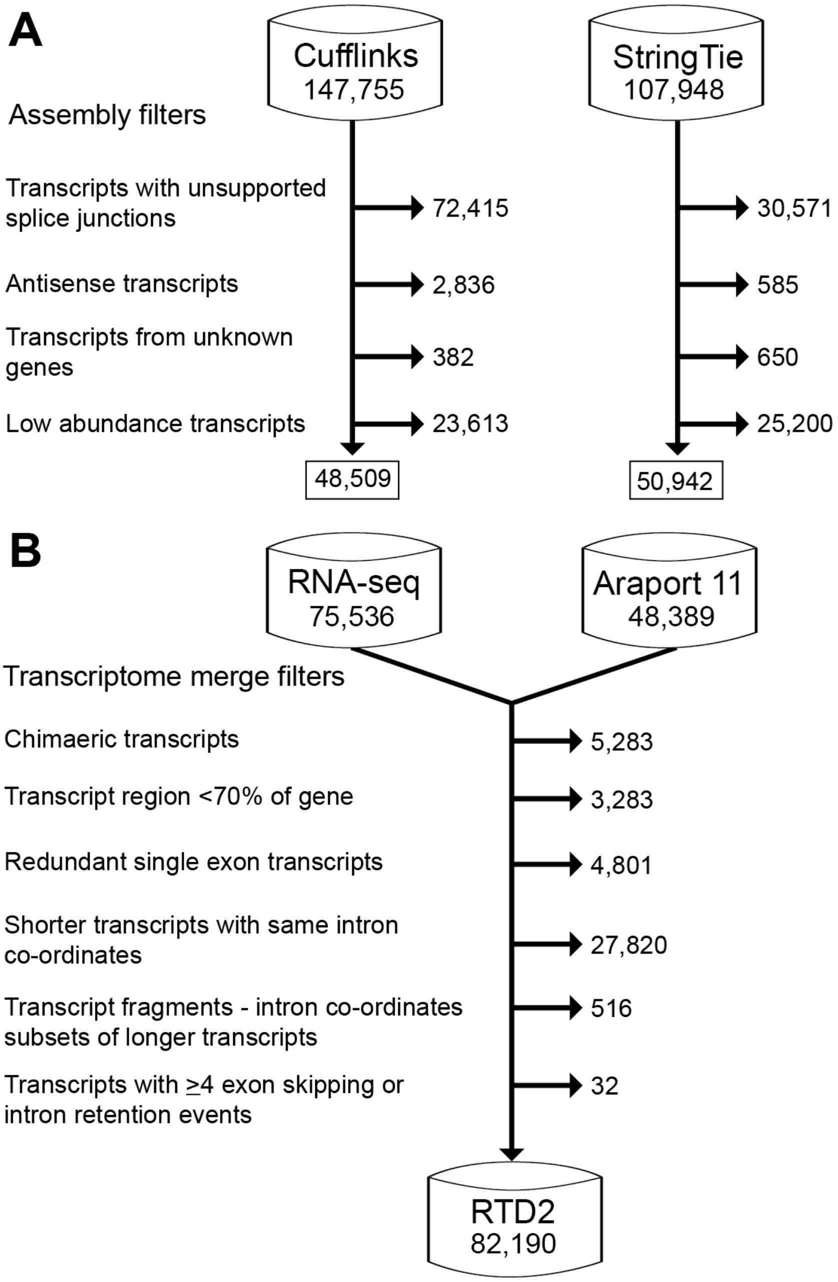

Figure S1. Pipeline of construction of AtRTD2: examples of application of quality and redundancy filters at the transcriptome assembly and transcriptome merge stages. (**A**) Dataset 1 was assembled by Cufflinks and StringTie. Transcripts unsupported by splice junctions, antisense transcripts, transcripts from unknown genes and transcripts with low abundance were removed. (**B**) The merged transcript sets obtained from the assembly and merge of RNA-seq Datasets 1 and 2 with AtRTD1 (“RNA-seq”) were merged with Araport11. Following the merge, transcripts were filtered on the basis of indicated criteria.

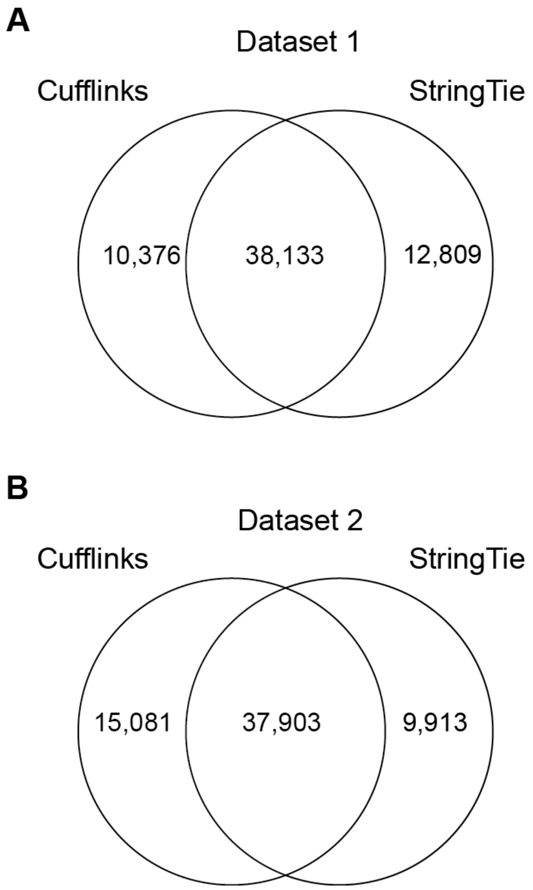

Figure S2. Overlap of transcripts assembled by Cufflinks and StringTie. (**A**) Dataset 1 and (**B**) Dataset 2. Datasets are described in Supplemental Methods below.

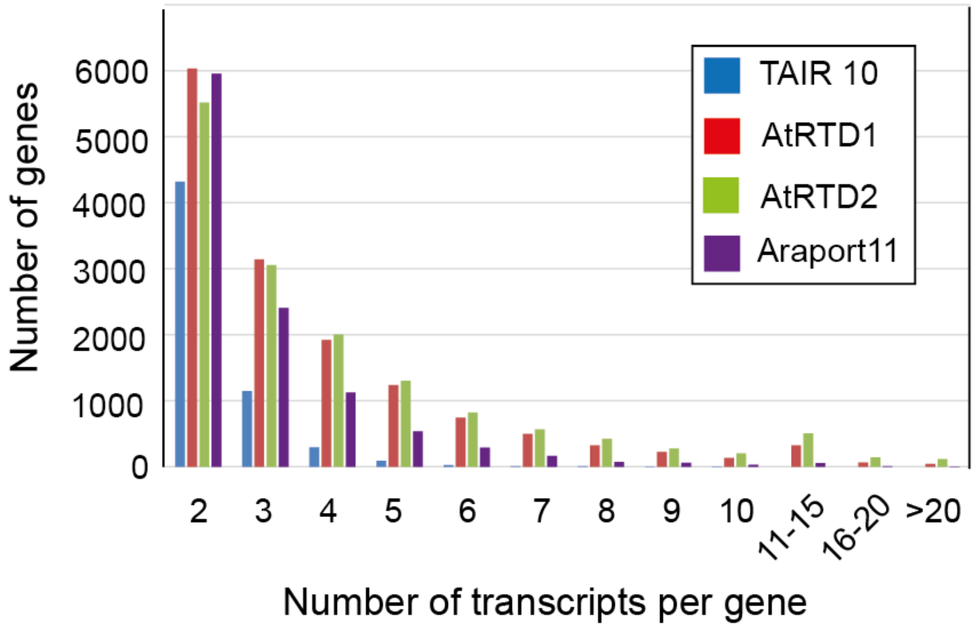

Figure S3. Distribution of the number of transcripts per gene in AtRTD2 compared to TAIR10, AtRTD1 and Araport11. The number of Arabidopsis genes (y-axis) containing two or more transcripts (x-axis) is shown. Figures are based on the total number of genes in TAIR10 (33,602), AtRTD1 (33,625), AtRTD2 (34,212) and protein-coding genes in Araport11 (27,667).

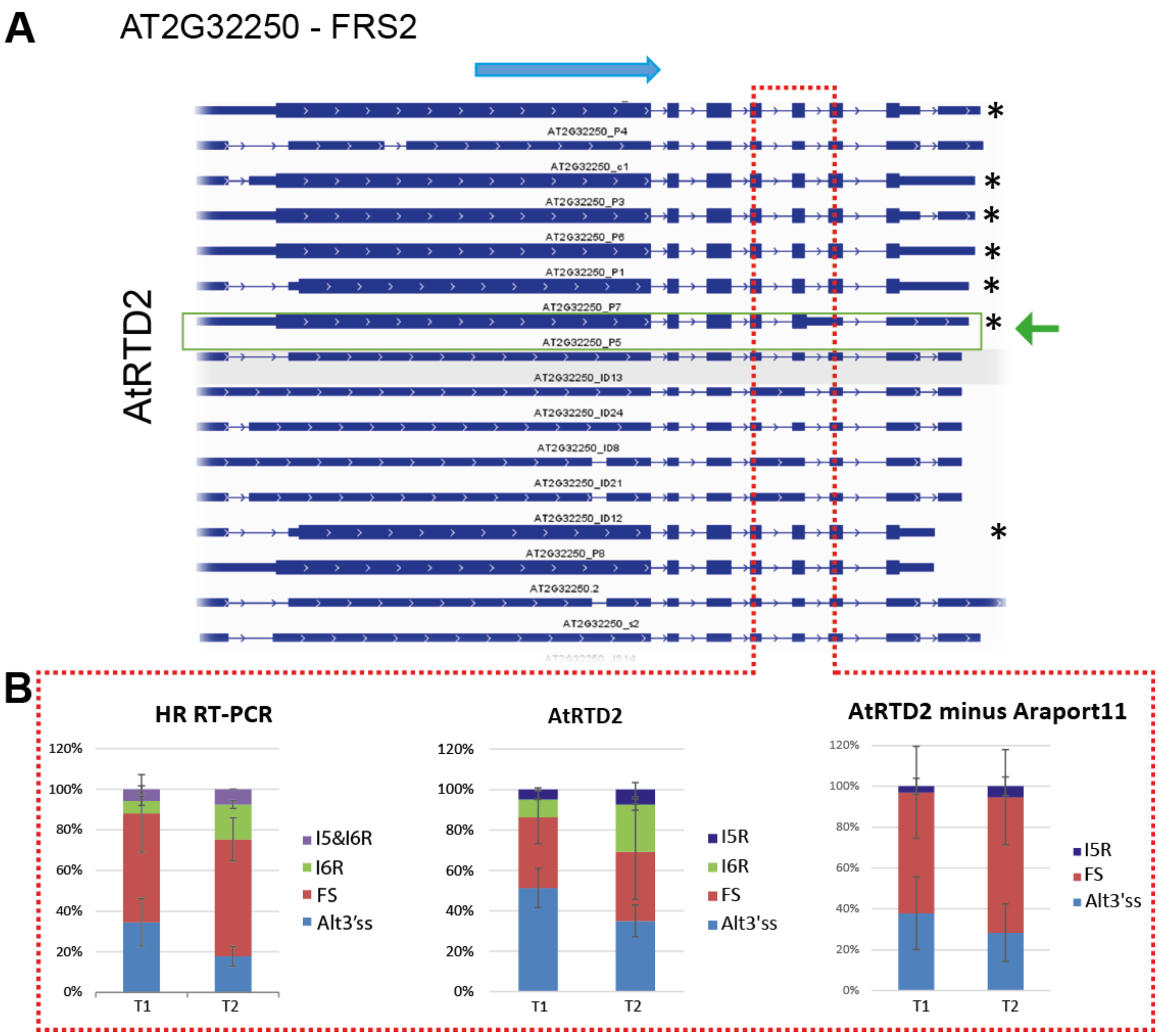

Figure S4. A complete transcriptome is important for accuracy of measurement of alternative splicing – *FRS2*. (**A**) *FRS2* (AT2G32250) has 17 transcripts in AtRTD2 which derive variously from TAIR10, Datasets 1 and 2, and Araport11. Seven isoforms are from Araport11 (asterisks). The region analysed using HR RT-PCR is boxed in a red dotted line. (**B**) The Araport11 AT2G32250.P5 transcript with retention of intron 6 (boxed in green) in particular impacts quantification of transcripts. Analysis of RNA-seq data with Salmon using AtRTD2 minus Araport11 transcripts (missing AT2G32250.P5) demonstrates the altered composition of AS events as shown by the ratio of different events when compared to AtRTD2 and HR RT-PCR. FS – fully spliced; I5R and I6R –retention of introns 5 and 6 respectively; Alt3'ss – alternative 3' splice site. Blue arrow – direction of transcription. T1 and T2 – time-points of Dataset 1 (see Methods).

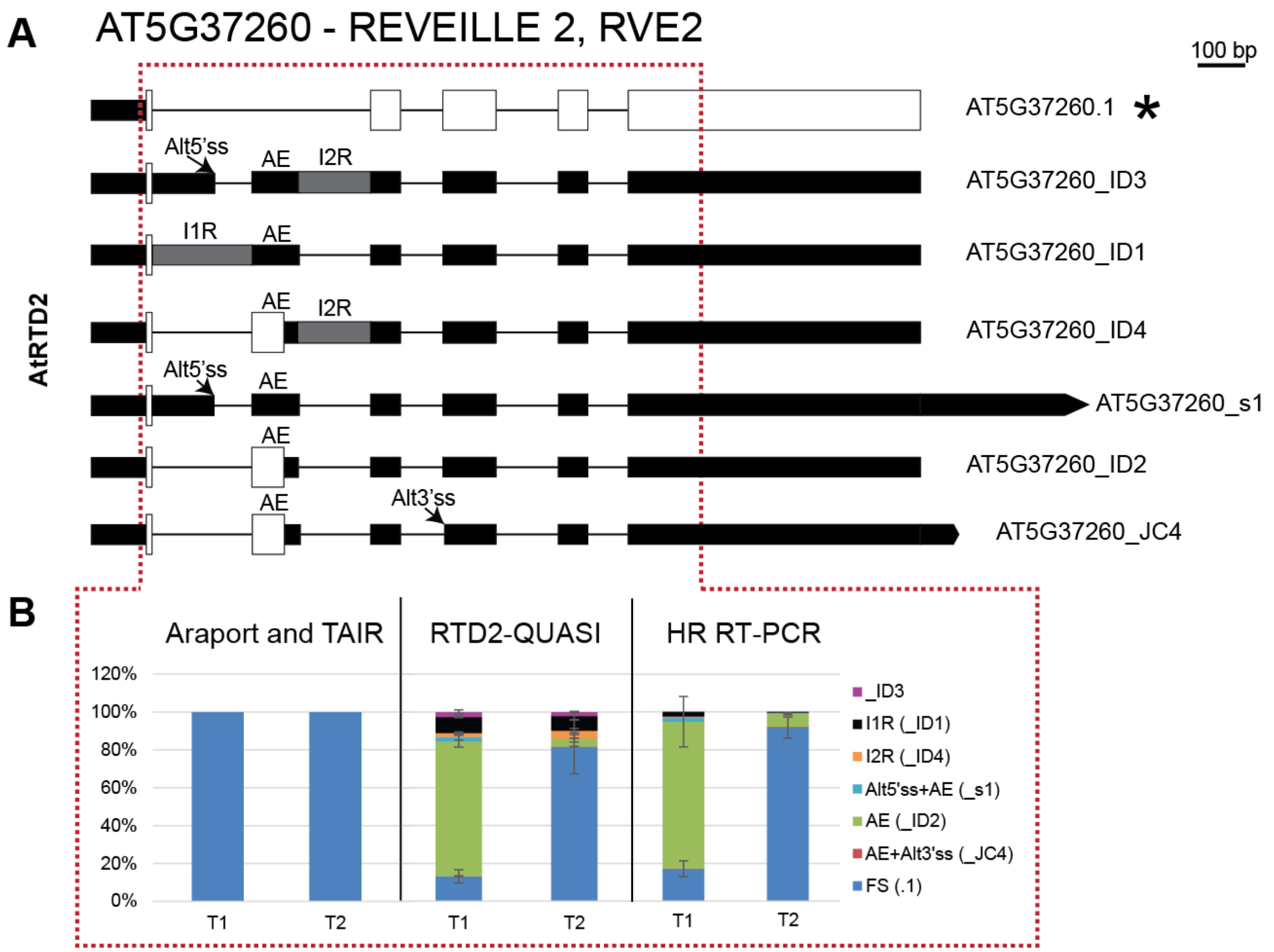

Figure S5. A complete transcriptome is important for accuracy of measurement of alternative splicing – *RVE2*. (**A**) *RVE2* has 7 transcripts but only one isoform is from TAIR10/Araport11 (asterisk). The region analysed using HR RT-PCR is boxed in a red dotted line. This region contains the Alt5'ss and AE in intron 1, intron 1 retention, intron 2 retention, and Alt3'ss in exon 4. For ease of description of these events, the AE is taken as exon 2 and the flanking introns as introns 1 and 2. (**B**) These events are absent in TAIR10 and Araport11 so that only the FS transcript is reported; however, analysis of RNA-seq data with Salmon/AtRTD2-QUASI generates splicing ratios which correlate well with HR RT-PCR. Transcript maps: white and black boxes – coding and non-coding sequences; respectively; grey boxes - intron retention. Error bars: standard deviation. Where an AS event causes introduction of a premature stop codon and loss of open reading frame, the downstream region is represented by black boxes (effectively a UTR) – these transcripts are classed as unproductive as they are not able to produce full-length protein (peptides or truncated proteins may be produced) and most transcripts are likely to be turned over by NMD. FS – fully spliced; AE – alternative exon; I1R and I2R – intron retention of introns 1 and 2 respectively; Alt5'ss – alternative 5' splice site; Alt3'ss – alternative 3' splice site; T1 and T2 – time-points of Dataset 1 (see Methods). See main text for description of AtRTD2-QUASI.

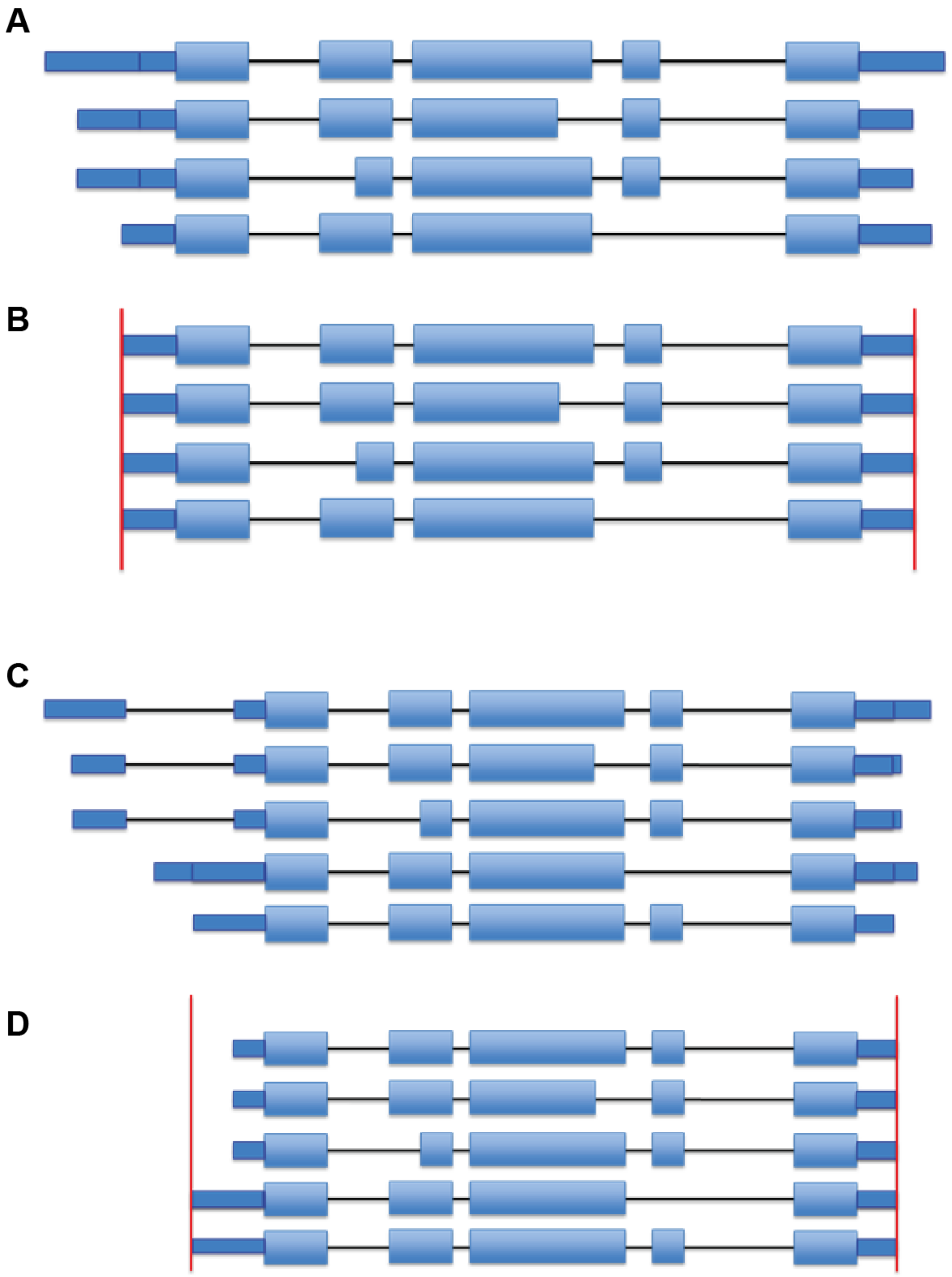

Figure S6. Trimming of transcript UTRs to similar lengths. (**A**) A gene with 4 transcripts with variation at the 5' and 3' UTRs. (**B**) Trimming (red line) to the co-ordinates of the ends of the shortest transcript makes all four transcripts have the same start and end co-ordinates (they can differ in length internally due to AS). In such cases (**A**, **B**) trimming improves the correlation of AS ratio to HR RT-PCR data. (**C**) A gene with five transcripts with variation at the 5' and 3' UTRs. The gene has an intron in the 5' UTR and two transcripts initiate within the intron. (**D**) Trimming (red line) to the co-ordinates of the ends of the shortest transcript generates new variation in 5' UTRs with the top three transcripts losing the first exon. In these cases (**C**, **D**), the AS ratios do not usually correlate well.

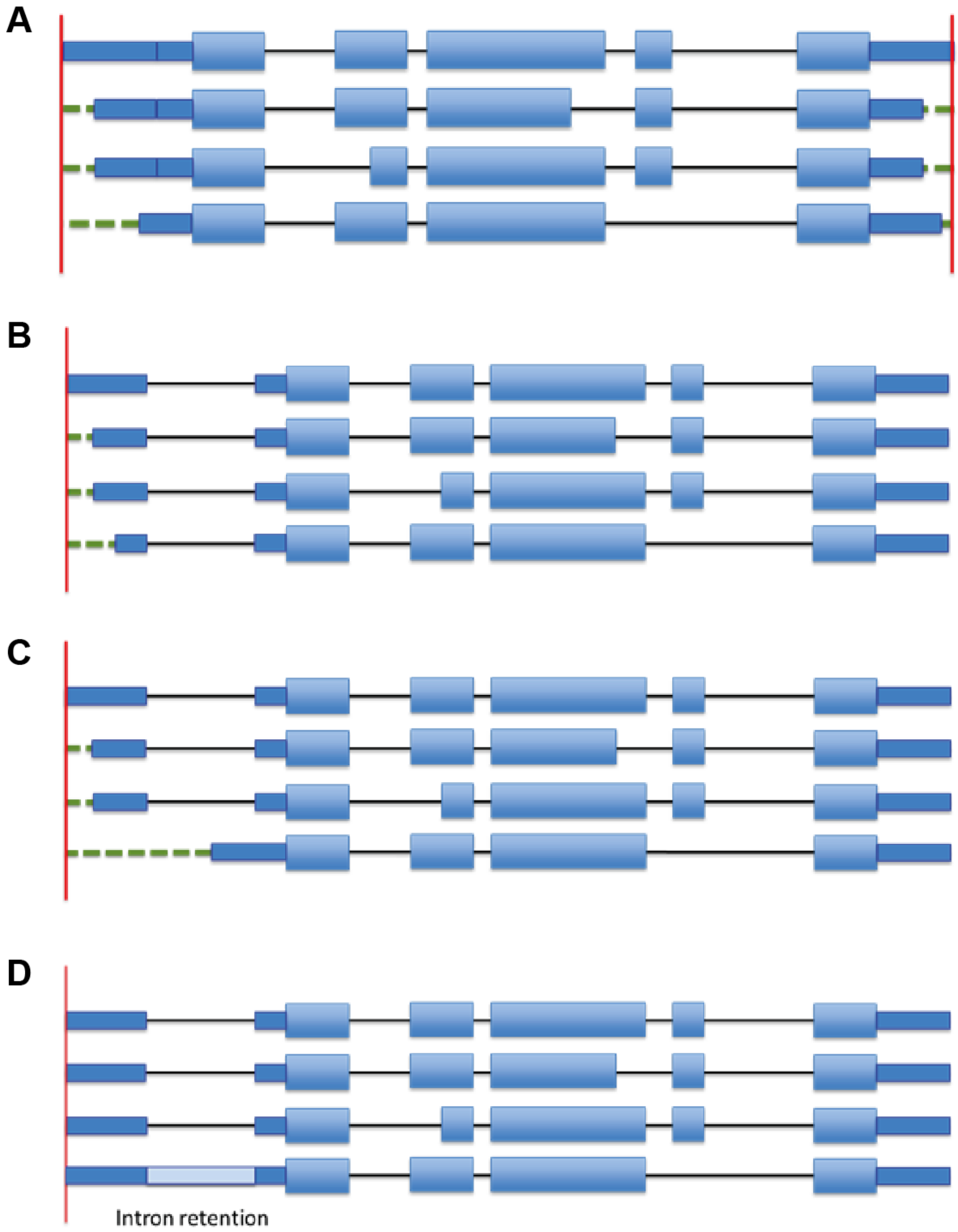

Figure S7. Padding of transcript UTRs to similar lengths. (**A**) A gene with 4 transcripts with variation at the 5' and 3' UTRs. Padding (green dotted line) to the end co-ordinates of the longest transcript(s) (red line) makes all four transcripts have the same start and end co-ordinates (they can differ in length internally due to AS). (**B**) As in (**A**), except the gene has an intron in the 5' UTR but here all transcripts have the intron spliced and padding makes the transcripts have the same start and end. In (**A**) and (**B**) padding improves the correlation of AS ratio to HR RT-PCR data. (**C**) The 5' end of one of the four transcripts is within the 5' UTR intron region suggesting that it is a possible intron retention transcript. (**D**) Padding to the end of the longest transcript with genomic sequence makes this transcript contain a retained intron.

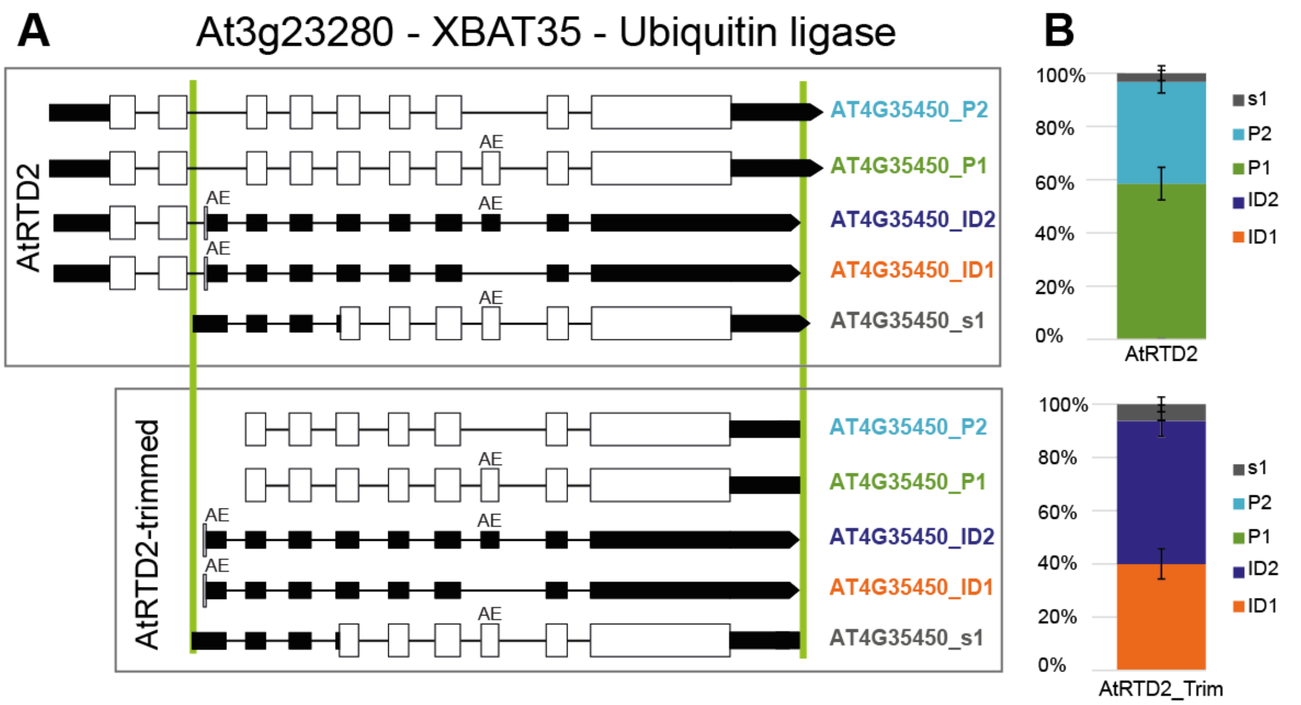

	TPM Salmon	
	**RTD2**	**Trimmed**
**ID1**	0.02	19.60
**ID2**	0.03	26.43
**P1**	28.13	0.01
**P2**	18.45	0.002
**s1**	1.55	3.02

Figure S8. Trimming of transcripts can generate new UTR length variation and affect isoform quantification of *XBAT35*. (**A**) AT3G23280 (*XBAT35*) has 4 transcripts with similar 5' ends and one shorter transcript whose 5' end lies in intron 2 (s1). Trimming transcripts to the shorter one (green line) removes exons 1 and 2 from the other transcripts causing s1 to now be the longest transcript and P1 and P2 the shortest. (**B**) Analysis of RNA-seq data from one time point in our experiment with Salmon using AtRTD2 and AtRTD2-trimmed demonstrates the effect of trimming on *XBAT35* levels as shown by the ratio of different transcripts in AtRTD2 compared to AtRTD2-trimmed. The table shows average TPMs of the 5 transcripts using Salmon and the AtRTD2 and AtRTD2-trimmed.

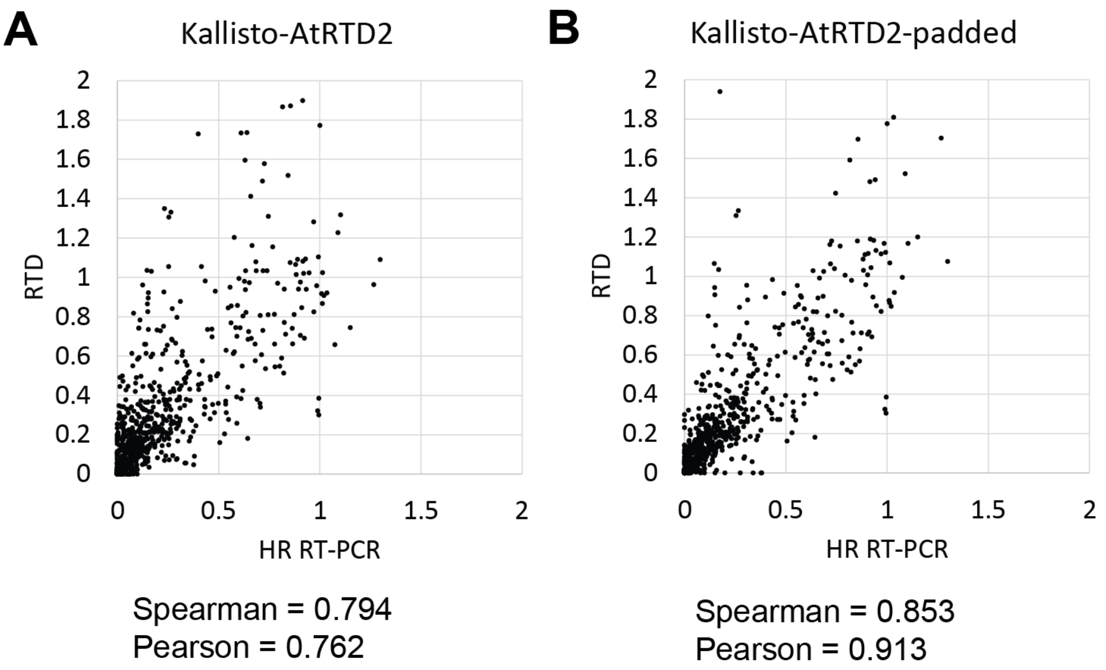

Figure S9. Correlation of the splicing ratios calculated from the RNA-seq data with Kallisto and HR RT-PCR. Splicing ratios for 127 alternative splicing events from 62 *Arabidopsis thaliana* genes (three biological replicates of the time-points T1 and T2 from Dataset 1, Table S1) generated 762 data points in total. The splicing ratio of an AS transcript to the fully spliced transcript was calculated from TPMs generated by Kallisto and (**A**) AtRTD2 and (**B**) AtRTD2-padded compared to the ratio from HR RT-PCR. Correlation coefficients are given with each plot.

A At5g05550 - *VFP5* trihelix-domain transcription factor

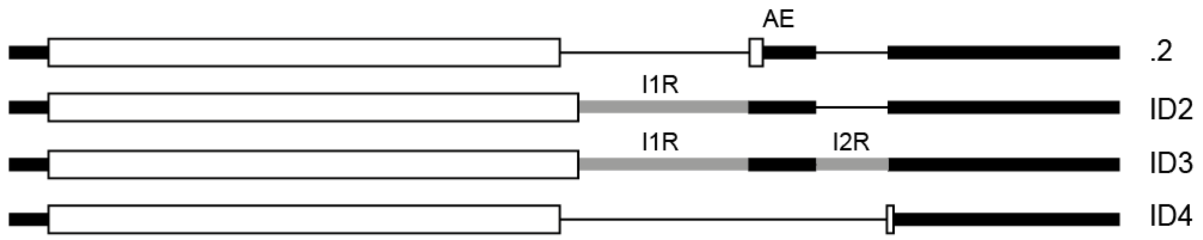

B

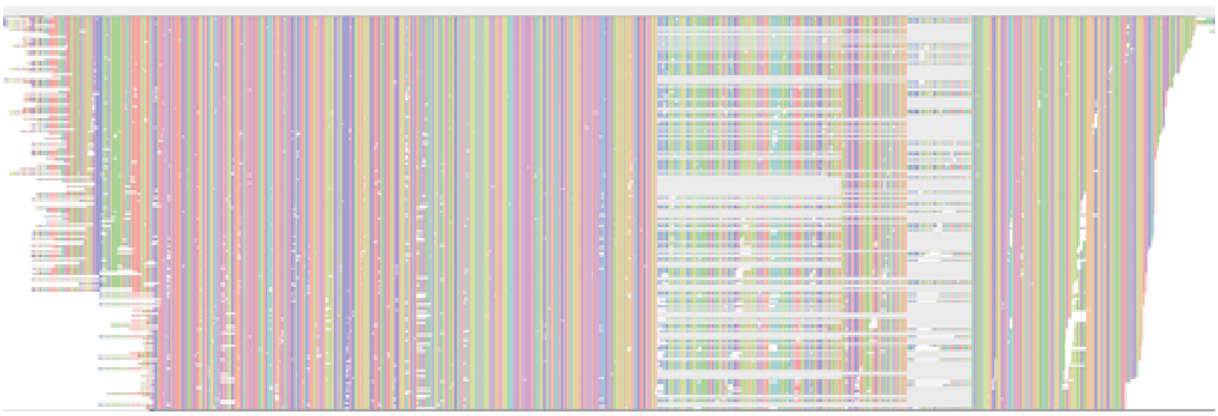

	TPM Salmon		TPM Kallisto	
	AtRTD2	Padding	AtRTD2	Padding
.2	6.15	6.41	5.82	6.00
ID2	5.76	5.95	6.36	6.59
ID3	2.55	2.60	2.90	3.00
ID4	7.03	7.35	6.28	6.49

	Ratio of transcripts with AE/FS (.2/ID4)
HR RT-PCR	0.63
Salmon-AtRTD2	0.87
Kallisto-AtRTD2	0.92
Salmon-padding	0.87
Kallisto-padding	0.92
Salmon-Araport11	1.00*

Figure S10. Transcript and AS quantification with no transcript variation in UTRs – *VFP5*. (**A**) *VFP5* (AT5G05550) has three different alternative splicing events (intron 1 retention – I1R; intron 2 retention – I2R and alternative exon inclusion – AE). Fully spliced (FS) transcript is represented by AT5G05550.ID4. The four transcripts have the same 5' and 3' UTR termini. (**B**) Screenshot of read alignment viewer showing splicie junction reads. The upper table shows average TPMs of the 4 transcripts obtained using Salmon and Kallisto and AtRTD2 and AtRTD2-padded; the lower table shows the splicing ratio of AE/FS obtained with HR RT-PCR, Salmon and Kallisto with either AtRTD2 or AtRTD2-padded, and Salmon with Araport11. *Note: Araport11 does not have the transcript containing both I1R and I2R (ID3) which could affect quantification of other isoforms.

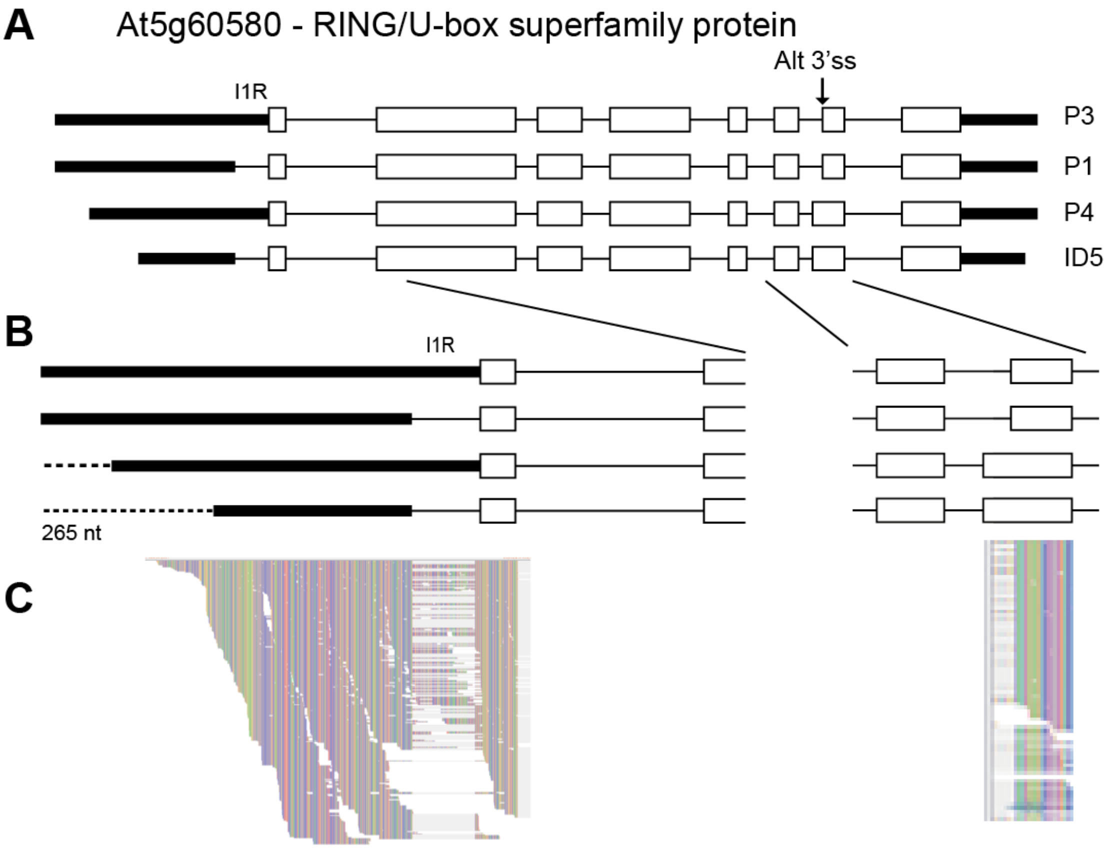

	TPM Salmon		TPM Kallisto	
	AtRTD2	Padding	AtRTD2	Padding
P3	2.80	2.29	3.26	2.72
P1	3.14	6.22	2.75	5.79
P4	0	0.45	0	0.53
ID5	4.25	1.08	4.29	1,07

	Ratio of transcripts with Alt3'ss/FS
HR RT-PCR	0.21
Splice junction read counts	0.19
Salmon-AtRTD2-padding	0.17
Kallisto-AtRTD2-padding	0.19
Salmon-AtRTD2	0.72
Kallisto-AtRTD2	0.72
Salmon –AtRTD2/ No Effective Length Correction	0.62
Kallisto –AtRTD2/ No Bias Correction	0.75
Salmon-Araport11	0.39

Figure S11. Transcript variation in UTRs affects transcript and AS quantification - RING/U-box superfamily protein. (**A**) AT5G60580 (RING/U-box superfamily protein) has two different AS events (intron 1 retention – I1R and an alternative 3' splice site, Alt3'ss, in frame in exon 8). (**B**) Padding of shorter transcripts – padding of ID5 transcript requires the addition of 265 nt. **c** Screenshot from read alignment viewer showing splice junction reads and that reads do not fully cover the longer transcripts. The upper table shows TPMs of the 4 transcripts obtained using Salmon and Kallisto and AtRTD2 and AtRTD2-padded; the lower table shows the Alt3'ss/FS splicing ratios obtained with HR RT-PCR, manual counting of splice junction reads in this region using the read alignment viewer Tablet, Salmon and Kallisto with either AtRTD2-padded and AtRTD2, Salmon and Kallisto with uncorrected functions with AtRTD2, and Salmon with Araport11.

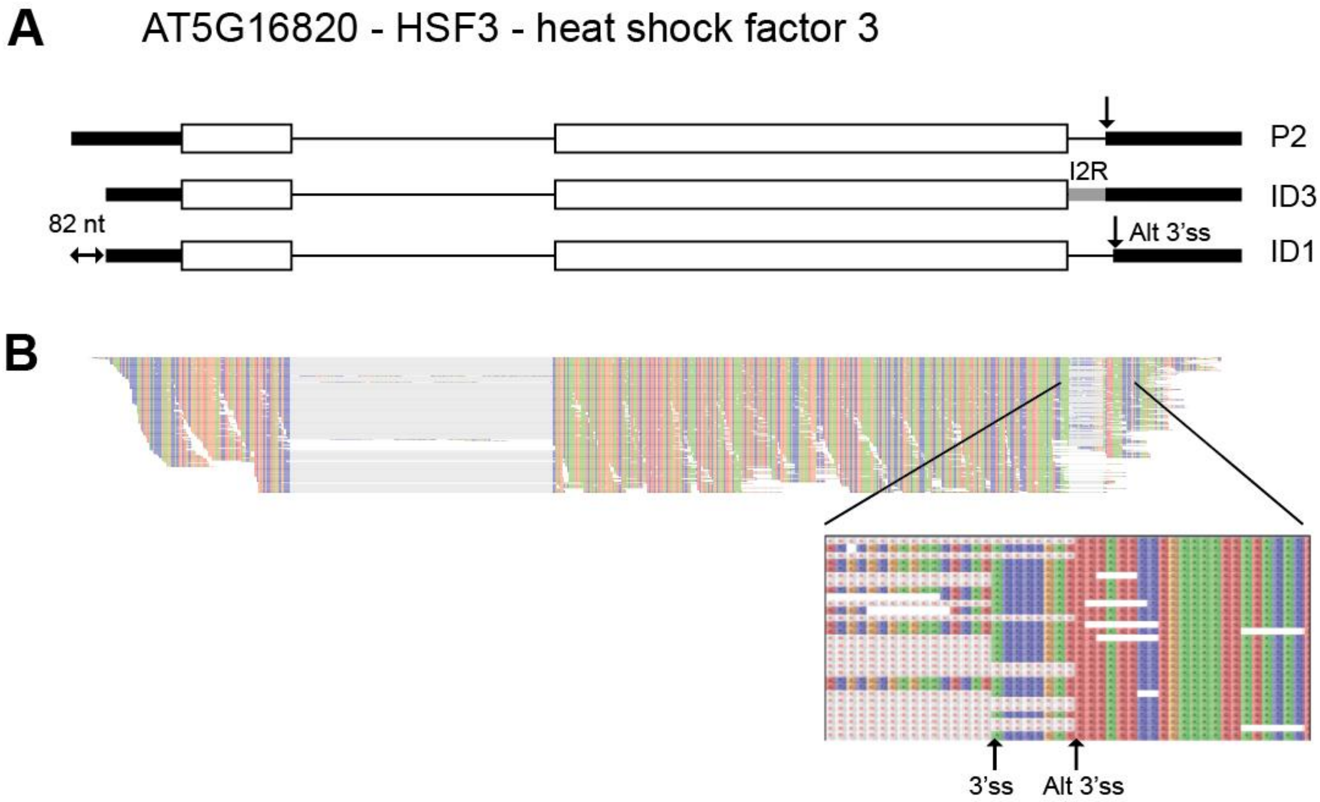

	TPM Salmon		TPM Kallisto	
	AtRTD2	Padding	AtRTD2	Padding
P2	1.36	1.68	1.38	1.64
ID3	1.12	1.07	1.24	1.02
ID1	5.23	4.97	5.20	4.96

	Ratio of transcripts with Alt3'ss/FS (ID1/P2)
HR RT-PCR	0.54*
Splice junction read counting	0.33
Salmon-AtRTD2-padding	0.35
Kallisto-AtRTD2-padding	0.34
Salmon-AtRTD2	0.26
Kallisto-AtRTD2	0.27
Salmon –AtRTD2/ No Effective Length Correction	0.28
Kallisto –AtRTD2/ No Bias Correction	0.26
Salmon-Araport11	0.31**

Figure S12. Transcript variation in UTRs affects transcript and AS quantification – *HSF3*. (**A**) AT5G16820 (*HSF3*) has two different AS events (intron 2 retention – I2R and an Alt3'ss which removes 8 nt). The P2 transcript has a longer 5' UTR by 82 nt. (**B**) Screenshot from read alignment viewer Tablet showing splice junction reads and magnified inset. The upper table shows TPMs of the 3 transcripts obtained using Salmon and Kallisto and the AtRTD2 and AtRTD2-padded; the lower table shows the Alt3'ss/FS splicing ratios obtained with HR RT-PCR, manual counting of splice junction reads in this region using the read alignment viewer Tablet, Salmon and Kallisto with AtRTD2-padded and AtRTD2, Salmon and Kallisto with uncorrected functions with AtRTD2, and Salmon with Araport11. * The HR RT-PCR splicing ratio value is over-estimated due to the presence of an artefactual peak which overlaps with the AS peak. ** Araport11 does not have the transcript containing I2R which could affect quantification of other isoforms.

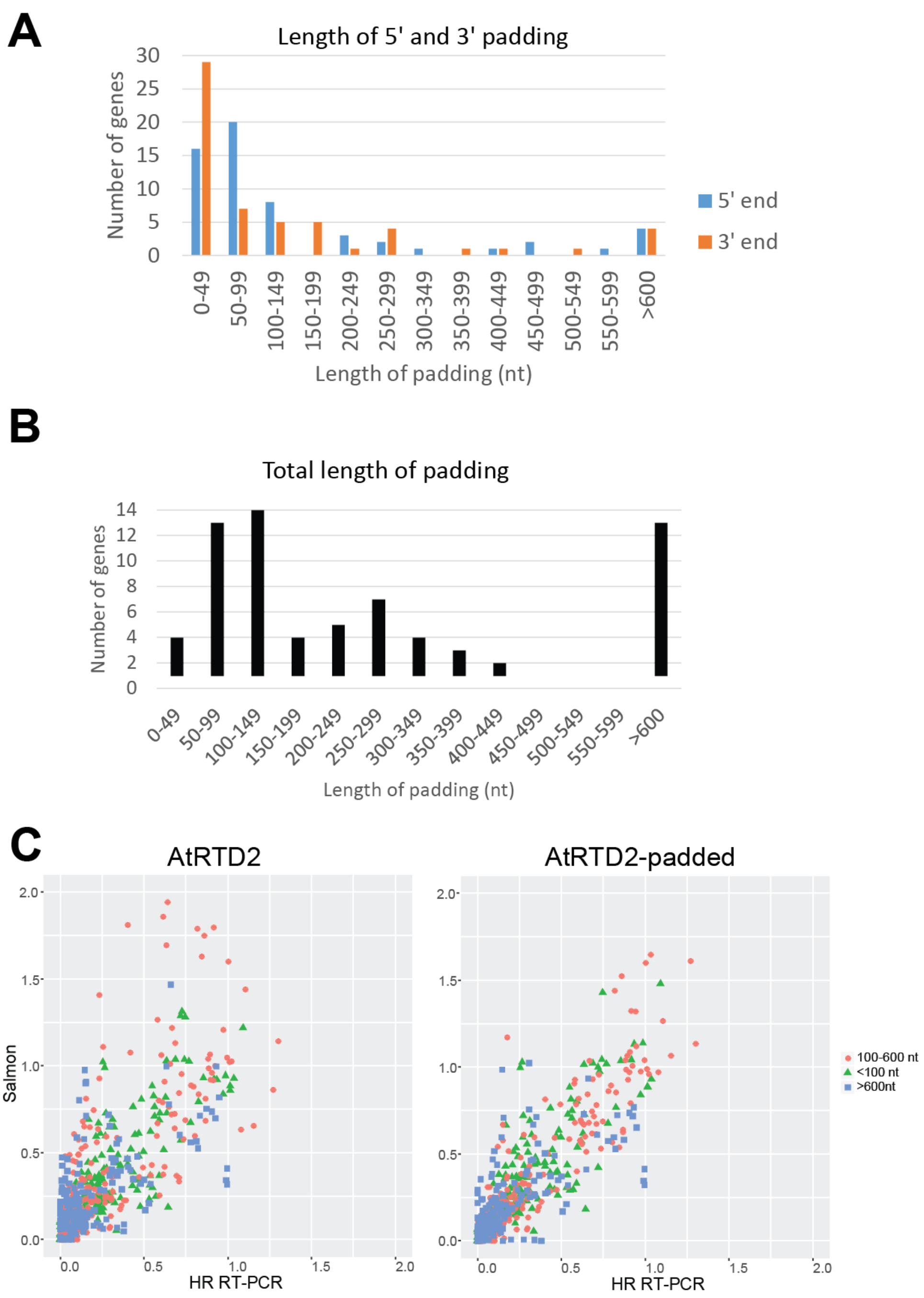

Pearson Correlation

	AtRTD2	AtRTD2-padded
**0-100 nt**	0.815	0.841
**101-600 nt**	0.718	0.971
**>600 nt**	0.612	0.676

Spearman Correlation

	AtRTD2	AtRTD2-padded
**0-100 nt**	0.843	0.863
**101-600 nt**	0.833	0.893
**>600 nt**	0.553	0.672

Figure S13. Effect of 5' and 3' UTR variation on accuracy of transcript and AS quantification. (**A**) The length of sequence added by padding to each transcript of the genes analysed by HR RT-PCR was determined separately for (**A**) the 5' and 3' ends and (**B**) both ends, and grouped into size classes. (**B**): 0-100 nt (green), 100-600 nt (red) and >600 nt (blue). Pearson and Spearman correlations are given for each size class. (**C**) AS/FS splicing ratios were calculated from TPMs generated by Salmon using AtRTD2 and AtRTD2-padded and compared to the ratio from HR RT-PCR. AS/FS splicing ratios were plotted by size class of the combined length of padding at the 5' and 3' UTR

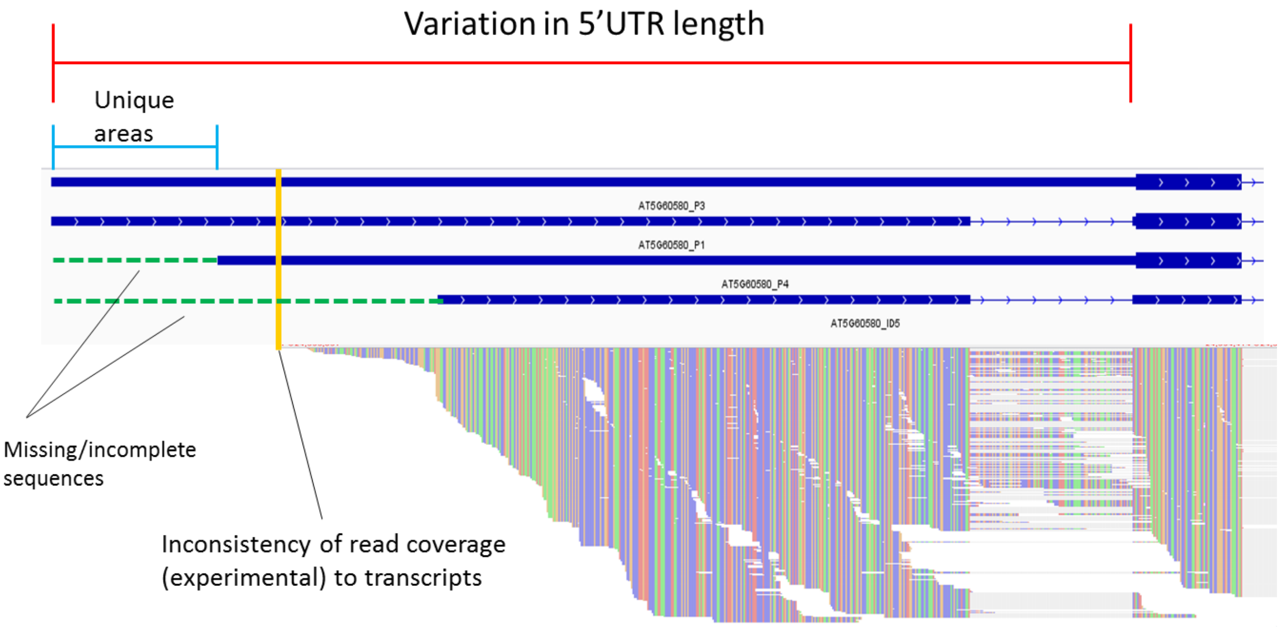

Figure S14. Potential issues affecting accurate transcript isoform and AS quantification. Examination of the transcripts of the genes analysed by HR RT-PCR shows different features of variation in 5' and 3' UTR lengths (shown here for the 5' UTR variation of a gene) which may influence quantification. In general, longer transcripts have sequences missing which are unique to them but missing in shorter transcripts, and often the reads from our experimental analysis do not cover the full length of the transcripts in the reference transcriptome.

Table S1. Arabidopsis samples used for RNA-seq to generate AtRTD2.

Line/treatment	Age of plants	No. of biological repeats
Dataset 1 (Dundee)		
Diel time-course at 20°C; sampled every 3 hours; 9 time-points	5 week old plants	3 per time-point
Diel time-course at 4°C after transfer from 20°C; sampled every 3 hours; 8 time-points	5 week old plants	3 per time-point
Diel time-course at 4°C, 4th day after transfer from 20°C; sampled every 3 hours; 9 time-points	5 week old plants	3 per time-point
Dataset 2 (Vienna)		
Normalised library (Marquez et al., 2012)	10 day old seedlings and flowers	1
At-*RS31* overexpression	10 day old seedlings	4
at-*rs31* mutant	10 day old seedlings	4
At-*RS2Z33* overexpression	10 day old seedlings	4
at-*rs2z33* mutant	10 day old seedlings	4
wild type control for SR protein lines	10 day old seedlings	4
met1-3 mutant	13 day old seedlings	3
wild type control for *met1-3*	13 day old seedlings	3
wild type + flg22	14 day old seedlings	3
wild type + mock	14 day old seedlings	3
mpk4 + flg22	14 day old seedlings	3
mpk4 + mock	14 day old seedlings	3
mpk3 + flg22	14 day old seedlings	3
mpk3 + mock	14 day old seedlings	3
mpk6 + flg22	14 day old seedlings	3
mpk6 + mock	14 day old seedlings	3

Table S2. Primer sequences used in HR RT-PCR [a].

Gene	Forward (5'→3')	Reverse (5'→3')
**AT1G33060**	GGCTAATTTAAGTGCTCAGGGG	GCTACATTTCGCATCACTTG
**AT5G16820**	GGAGAATAATGACTTGGTATTGG	GGTAAATCTTTTATGTTTCTTC
**AT2G32320**	CTTTTGGAATGAGCACTCC	CCGTAGGCAAAGGCAATATCC
**AT3G01150**	CCATGAGATTGTTAACAATCAGAGTCC	CCAGCAGCTTTCTCAAATGTGGC
**AT1G04400**	TTTGTCGCCGAGAGATA	CAATCCAAGATCGCTGAA
**AT5G48150**	CCTTGTCTCCGACAACTTTC	CCTAAGCTTCTCAACAGAGTTAG
**AT5G09230**	CTCCAAGGTTTCTGAGGGATAG	CGTAACTCCTCGCCCAATAC
**AT5G09230**	GGACCAGCTCATACTGCTTTAG	ATCAGGCTTTAGGACTCCTTTG
**AT5G09230**	GACAACATCCCGAAGGAAAGA	ACGTCAAGAACTCTGTGCAATA
**AT5G04430**	GAGTCATATGCGGCAGGTTC	ACCAGCTTTAGATTCCTCAATGA
**AT1G69250**	CGCTGCTCCGTTTCAAGTTA	CCATTCTCAGTCTTGCTGCTATC
**AT2G39730**	CCTCCCGTGTTCGAGCAACCC	CCGTTGGATCAAAGTTTTCAGCC
**AT5G18830**	GGAGCCAGACAGTCTTGTTCAC	GGATCAGTCTCTTTTCCGCC
**AT3G49430**	CCTCCGAGTATTGTTGGCTTCAGACC	CCTAATGTCACCGGGCAAGTTACC
**AT4G16420**	GCAGAACATGAAAGAAGAGTAC	GCTCAGCATCATTGTCATATTC
**AT1G33470**	CACTTCCATTCTCCCACTACAC	GGAACTATGAACCACGAGCC
**AT1G33680**	AAAGGTGGGGAGATGGTACG	CCTCGAGGTTGGTAAGCC
**AT1G77180**	GCTTCACGCCCGATTTCTTT	GAGAGTCTTAGCTCCAGGCTTATT
**AT4G26650**	GATCCCTGGCAACCAATACTT	ACAATTCTCACTTCTCTTCGTCG
**AT5G43960**	TCCCTTCTTCTCTGTAAACCCT	TCCCACGACTCGACTGAATTA
**AT5G66010**	AACGAACCAGGGAACAAGAA	GCACGTACCTCATACTCATACG
**AT3G12250**	GGACAACCTTTTACTCAGACAGG	GGTATCAGCCATACTAGTTTCTG
**AT3G12250**	CCCTCTAGTGTGAAACTCTGC	CCTTTGATCGATCACTGGAATC
**AT2G32250**	CCCATCGATTTCTTCCATCC	CTTCGATGTCATAGCAGCC
**AT1G01060**	CCCGGTGAGATGATAAGTC	CCATCTTTGATCTCCCCAAAC
**AT1G01060**	GGGACAAAGACTGCTGTTCA	CAGGCTTTCGAGGATAAGGA
**AT1G30200**	GGAACCGTTTGGGACTGTG	GCAGATTCATTCGACGCAGG
**AT1G59750**	CCGTCTTCACAACCTCAGCC	CCCACCATGACCAAACGTAGC
**AT1G72050**	GGTTGATGAGGAGTCTTCAAG	GGACACTTAAAGAGCTTCCC
**AT1G72650**	GGTTTATTGCATTCTGATAATG	GGTGGACTTGATCCTCCTC
**AT1G76510**	CCGTTTCTCGCTTCTTTTTCTC	CCTCTACAACACCTTTGGTACC
**AT2G18300**	GGATGCAATAAGGTCACAGG	GGTCTAACGGAAACAATGG
**AT2G36010**	CCAAAGATGGAATGCTGGACC	CTCAGGTCTCTTAATCTTTCC
**AT2G38880**	GGCAACATTAGGATTTGAGG	GGAAACAATAAACCAAACG
**AT3G23280**	GCACAGTGATGCCTTTGTGG	GGCTGTCACCTTCAGTCGAAGG
**AT3G51880**	GGGAGTGATGAATCTGAAAAG	GGATCTGCAGTTAAGCTTGAG
**AT3G54230**	TAAAGTTGGATTCGGCCAAG	TGTTATTATCCCATTCTTGCTGA
**AT1G22330**	ATCAAGGCGGAGGACAATCA	GATGGAGTAGGAGCCTCGAC
**AT4G10070**	CCAGATGACGGAGGAAGATTT	CCACCTTTACCAATCAACACAC
**AT4G10070**	CAACCTGGTGTTAGGCCTTATG	GGCTTGCATCGTATTGAGCA
**AT4G01060**	GGTGCCGTTTGACATGGATAACC	GTCACCGACAAGCTTATGCATTC
**AT4G32730**	CTCAGCGTCTTAATTACTTCAG	CCTTGGAGACTCTCTAGTGGAG

**AT4G35450**	CCACCACAACATTGTCTTTTC	CCAGCGTTAGGAATAGATCTC
**AT5G05550**	GGAGAAGCAGAGAATGGAAG	GGATCCTCCAATTTCAATGAG
**AT5G06960**	CCACGTCTTTGCAGTTGTAG	CCATCTGTTGAGACTGATGTTC
**AT5G12840**	GGATTGATGGGAGCATATGG	GGCACGTGCTTTTCTTCGCC
**AT5G13220**	CCCATCGCAAGGAGAAAGTC	CCAAATCCAAAAACGAACATGG
**AT5G41150**	CCATCCTGACATGGGTTTTGTC	CCAGTTCCTTTCTTCCGCCTGC
**AT5G18620**	CCATCAAAACGACCTTCGGG	CCTTGTCTCTAAGGAGCTTC
**AT5G43910**	GGTTGTGTGATGCTTGAGAG	GGTTGTGTCTATGAGTTCCG
**AT5G60580**	GGTACAGGTGCCATCGCTATATC	GGGCGAAGAGAACGACCAACGC
**AT4G15090**	TTCCTCACTCGCTTTTGCTT	CATCCCCTGCATCACTAACC
**AT3G22170**	CGGCCTTTTCTCAGTTTCTG	GGAACACCCATGCCTACACT
**AT3G22170**	CTGTGGAGGAGGACAACCAT	TGCAACTGCGTAGTCCTCAC
**AT2G21070**	TTCGGTTCGTTGGAAGAAAG	CCCACATGCAATGCAAGATA
**AT3G17609**	TGTCTCTCCAACGACCCAAT	CTTCGAGCTGGTCATTGTTG
**AT1G09530**	ACGATTTGGTCCCCTCTCTT	AAGGGTTCCTGTCTTGAGCA
**AT5G35840**	GGTGCAAGACACGATCCTAAT	AACCGGACTGCTTTTCCTTT
**AT5G61380**	TGTGGACACACATGTGGAGA	TTCGGTGCTGAAGAAAATTG
**AT3G59060**	TGAACCTGGCAAGAAGAACC	TTGATCTTTTATTAGTGGACATGTGA
**AT3G59060**	CCACTACACTTCCGGTTTCTATG	CGAGCTGCTCCGATAAGATTT
**AT5G02840**	TCAAACCCTGACTTTGAACCT	TTGTGACGACGACTCAGTTATG
**AT1G01520**	AGAACTGGACGGAGCAAGAA	GCTTACGGGTGGTGCTAATC
**AT2G20180**	GACCGCAATACCCTGTTCAT	GCACTAAGCCTCTGCAACCT
**AT2G20180**	TGCATCATTTTGTCCCTGAC	TTCACCACCTCTACCGTTATT
**AT5G13730**	GGGTACCAAGGCAAAGGTTTG	GCAATCTCTTCACAGCTTGG
**AT5G60100**	ATTCTGGTGGTGGTGGTGTT	CAAACACTTGCCTCTCGGTT
**AT5G37260**	GCAACCATGGCTATGCAG	CCGATGTTGGAGATCGGTTA

[a] Primer pairs were selected based on previous data providing AS events that ranged from 10-50% of the total transcripts.

Table S4. Alternative splicing events occurring at least 50 times in AtRTD2 according to AStalavista.

Rank	Intron-exon structure	Event description	Events	Freq. (%)	IR
**1**	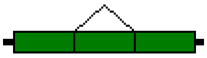	Intron retention (IR)	10,272	27.66	+
**2**	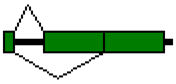	Alternative 3' splice site (Alt 3'ss)	9,264	24.95	
**3**	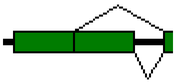	Alternative 5' splice site (Alt 5'ss)	4,133	11.13	
**4**	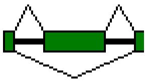	Exon Skipping (ES)	2,120	5.71	
**5**	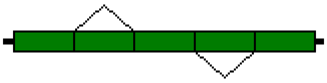	IR1 *or* IR2	1,592	4.29	+
**6**	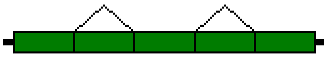	IR1 *and* IR2	1,518	4.09	+
**7**	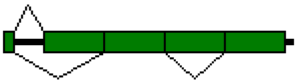	Alt3'ss *and* IR	765	2.06	+
**8**	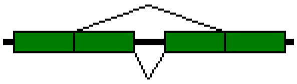	Alt 5'ss Exon 1 (E1) *and* Alt3'ss E2	737	1.98	
**9**	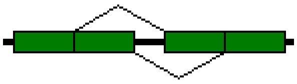	Alt 5'ss E1 *or* Alt3'ss E2	715	1.93	
**10**	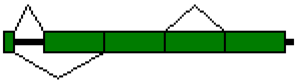	Alt3'ss *or* IR	707	1.90	+
**11**	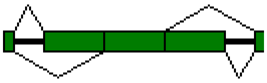	Alt 5'ss *or* Alt3'ss	316	0.85	
**12**	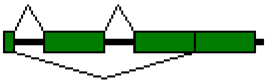	ES and Alt3'ss	309	0.83	
**13**	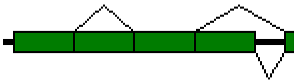	Alt5'ss *or* IR	307	0.83	+
**14**	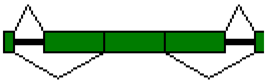	Alt 5'ss *and* Alt3'ss	279	0.75	
**15**	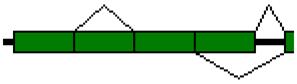	Alt5'ss *and* IR	252	0.68	+
**16**	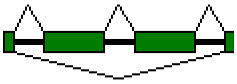	ES1 *and* ES2	243	0.65	
**17**	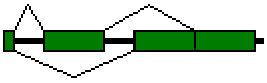	ES or Alt3'ss	224	0.60	
**18**	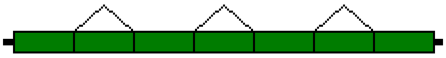	IR1, IR2 *and* IR3	222	0.60	+

Rank	Intron-exon structure	Event code	Events	Freq. (%)	IR
**19**	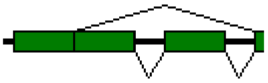	Alt5'ss *and* ES	188	0.51	
**20**	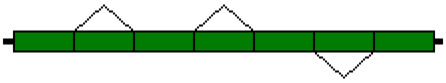	IR1 *and* IR2 *or* IR3	171	0.46	+
**21**	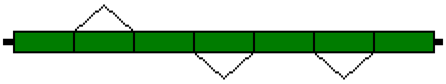	IR1 *or* IR2 *and* IR3	160	0.43	+
**22**	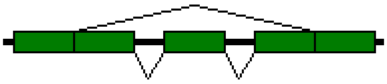	Alt5'ss, Alt3'ss *and* ES	145	0.39	
**23**	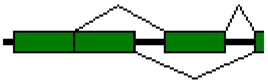	ES *or* Alt5'ss	144	0.39	
**24**	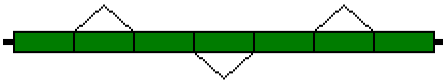	IR1 *and* IR3 *or* IR2	124	0.33	+
**25**	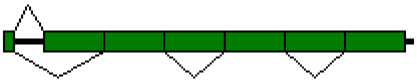	Alt3'ss *or* IR1 *and* IR2	111	0.30	+
**26**	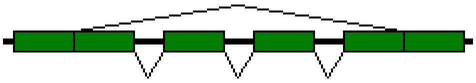	Alt 5'ss, ES1, ES2 *and* Alt3'ss	99	0.27	
**27**	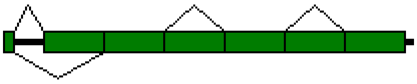	Alt3'ss, IR1 *and* IR2	83	0.22	+
**28**	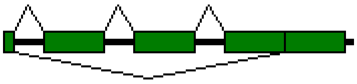	ES1 *and* ES2 *or* Alt3'ss	61	0.16	
**29**	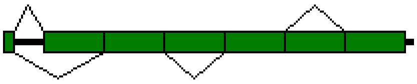	Alt3'ss *and* IR2 *or* IR1	56	0.15	+
**30**	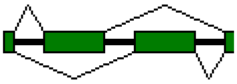	Mutually exclusive exons	51	0.14	

Supplementary Methods: Pipeline for generation of AtRTD2

Transcript assembly of Dataset 1

RNA-seq reads were generated for three biological repeats and three sequencing repeats on different lanes for each of the 26 time-points (78 libraries, 234 total biological/sequencing repeats) (Table S1; Figure 1B). Read mapping to the genome was performed with STAR on individual biological/sequencing repeats with a 2-pass mapping to allow more splice junction reads to map to novel junctions. The parameters of the mapping were set as the following:

- --sjdbOverhang 100
- --outSAMprimaryFlag AllBestScore :*output all alignments with the best score as primary alignments*
- --outFilterMismatchNmax 2/0(first/second pass): alignment will be output only if it has fewer mismatches than this value
- *--outSJfilterCountTotalMin 10 5 5 5 (non-canonical SJ and 3 canonical SJs)*
- --outSAMstrandField intronMotif
- --outFilterIntronMotifs RemoveNoncanonical : *filter out alignments with non-canonical junctions*
- --alignIntronMin 60 : *mininum intron size*
- --alignIntronMax 6000 : *maximum intron size*
- --outSAMtype BAM SortedByCoordinate: *output sorted BAM file*

Reads were mapped using intron length range parameters of 60 nucleotides (nt) (minimum intron length) to 6000 nt (maximum intron length) (Marquez et al. 2012) and allowing 2 mismatches for the 1st pass and no mismatches for the 2nd pass. Transcripts were assembled for each library/replicate in parallel using Cufflinks (Trapnell et al. 2010) and StringTie (Pertea et al. 2015) in the Reference Annotation Based Transcript (RABT) mode with the AtRTD (Zhang et al. 2015). Assemblies were first merged with Cuffmerge to give two transcriptomes generated by Cufflinks and StringTie (Figure 1B). Each transcriptome was filtered to remove unknown genes, antisense transcripts and transcripts which were poorly supported by splice junction reads (i.e. transcripts containing one or more splice junctions which had fewer than 10 splice junction reads in less than 3 samples). In addition, only transcripts which had TPM (transcripts per million) values of >1 in at least three sequencing samples were kept. Finally, the resulting Cufflinks and StringTie transcriptomes were merged into a single transcriptome with in house scripts. The final transcript assemblies were then filtered to remove redundant transcripts (redundant single exon gene and alternatively spliced transcripts with the same intron co-ordinates but different transcript lengths), keeping the longer transcripts. This generated a transcriptome from the diel/temperature series of 57,732 transcripts (Figure 1B).

Transcript assembly of Dataset 2

RNA-seq reads were generated from 51 various libraries listed in Table S1. Read alignment was carried out with TopHat2 (Kim et al. 2013) using intron length range parameters of 60 nt (minimum intron length) to 6000 nt (maximum intron length) (Marquez et al. 2012) and splice junction reads were anchored by allowing no mismatches in the first 12 nt of the flanking exons. Alignments for each of the biological repeats was

performed independently and assembled with Cufflinks and StringTie using TAIR10 genes as a reference annotation. The resulting transcriptomes (i.e. for each biological repeat) were merged with Cuffmerge to generate a transcriptome for each biological sample. The different Cuffmerge files were then all merged together to generate two total transcriptomes assembled with Cufflinks and StringTie (Figure 1C). The assemblies were filtered to remove transcripts from unknown genes, antisense transcripts, and transcripts unsupported by splice junction reads or with low expression as described above. The resulting two transcriptomes were then merged to generate a transcriptome of 62,897 transcripts. To simplify the next merge, the transcriptome was compared to TAIR10, and TAIR10 transcripts were removed along with redundant transcripts (as above). In addition, due to our focus on alternative splicing, we have added transcript isoforms for the SR family of splicing factor genes (for which there is experimental evidence but not all were assembled) to the final transcript set. Redundant transcripts were then removed to give 20,019 transcripts (Figure 1C).

Merge of AtRTD1 with Dataset 1 and 2 transcriptomes

The original AtRTD (now referred to as AtRTD1) was generated from merging the 41,671 transcripts from TAIR10 and the 57,408 transcripts from Marquez et al. (2012) and removing redundancy (Zhang et al. 2015). To further quality control AtRTD1, the splice junction read data from the assemblies of Datasets 1 and 2 were mapped to AtRTD1. A total of 12,842 transcripts contained unsupported transcripts of which 5,610 were derived from TAIR10 and 7,232 from Marquez et al. (2012). The latter were removed because they most likely represented mis-assembled transcripts while the TAIR10 transcripts were retained. The modified AtRTD1 was then merged with the Dataset 1 transcriptome by first merging the gene models keeping the longer models, separating any overlapping gene models keeping individual models, and extracting exact intron co-ordinates from the two annotations. The merged transcriptome was filtered as described below. The resulting transcriptome of 69,089 transcripts was then merged with the Dataset 2 transcriptome (20,019 transcripts) and the same filters as below were applied, generating 77,356 transcripts.

Splicing/transcript quality filters to remove redundancy and minimise unlikely transcripts

Transcripts from unknown sources (not associated with a known gene), antisense transcripts and chimeric transcripts were removed. For the latter, the fused gene models were separated and transcripts associated with the separate genes were retained. The presence of redundant transcripts affects transcript quantification. Therefore, redundant transcripts which had the same intron co-ordinates but differed in the length of their 5' and/or 3' UTR lengths were removed as well as redundant single exon transcripts with different UTR lengths (longer transcripts being retained). Transcript fragments, generated by RNA-seq when genes have insufficient read coverage, were removed if they contained the same intron co-ordinates of longer transcripts (i.e they were subsets of longer transcripts) because they interfere with accurate quantification. In addition, transcript fragments which covered less than 70% of the longest transcript of a gene were removed irrespective of intron co-ordinates as they also affect accuracy of quantification. This removed, for example, around 5% of transcripts from the Dataset 1 transcriptome. In our alternative splicing analyses, we identified examples of transcripts from multi-intron genes which contained two or more retained introns or which skipped two or more exons (Kalyna et al. 2012; Marquez et al. 2012). RT-PCR evidence of transcripts for particular genes with two

skipped exons or two retained introns suggested that such transcripts are real (e.g. AT5G24270 and AT4G24740 (AtAFC2) respectively – (Kalyna et al. 2012; Marquez et al. 2012)). However, it is highly likely that transcripts containing multiple retained introns represent partially spliced intermediates. It is unlikely that alternative splicing events which remove multiple exons and thereby large stretches of sequence are real. Therefore, we have conservatively removed transcripts that have four or more consecutive retained introns and four or more consecutive skipped exons.

Merge with Araport11 Pre-release 3 transcripts

The Araport11 Pre-release 3 (December 2015) transcript set contains 48,389 transcripts and includes new genes and transcripts and has extensively increased the 5' and 3' UTR regions of nearly 70% of the protein-coding genes in comparison to TAIR10 (Cheng et al. 2016). This transcript set was merged to the 77,356 transcripts from above and the same filters were applied. The resulting Arabidopsis transcriptome, AtRTD2, contains 82,190 non-redundant transcripts.

Supplementary References

Cheng C-Y, Krishnakumar V, Chan A, Schobel S, Town CD. 2016. Araport11: a complete reannotation of the Arabidopsis thaliana reference genome. *Plant Journal* **doi: 10.1111/tpj.13415**.

Kalyna M, Simpson CG, Syed NH, Lewandowska D, Marquez Y, Kusenda B, Marshall J, Fuller J, Cardle L, McNicol J et al. 2012. Alternative splicing and nonsense-mediated decay modulate expression of important regulatory genes in Arabidopsis. *Nucleic Acids Research* **40**(6): 2454-2469.

Kim D, Pertea G, Trapnell C, Pimentel H, Kelley R, Salzberg SL. 2013. TopHat2: accurate alignment of transcriptomes in the presence of insertions, deletions and gene fusions. *Genome Biology* **14**(4): R36.

Marquez Y, Brown JW, Simpson C, Barta A, Kalyna M. 2012. Transcriptome survey reveals increased complexity of the alternative splicing landscape in Arabidopsis. *Genome Research* **22**(6): 1184-1195.

Pertea M, Pertea GM, Antonescu CM, Chang TC, Mendell JT, Salzberg SL. 2015. StringTie enables improved reconstruction of a transcriptome from RNA-seq reads. *Nature Biotechnology* **33**(3): 290-295.

Trapnell C, Williams BA, Pertea G, Mortazavi A, Kwan G, van Baren MJ, Salzberg SL, Wold BJ, Pachter L. 2010. Transcript assembly and quantification by RNA-Seq reveals unannotated transcripts and isoform switching during cell differentiation. *Nature Biotechnology* **28**(5): 511-515.

Zhang R, Calixto CPG, Tzioutziou NA, James AB, Simpson CG, Guo W, Marquez Y, Kalyna M, Patro R, Eyras E et al. 2015. AtRTD – a comprehensive reference transcript dataset resource for accurate quantification of transcript-specific expression in Arabidopsis thaliana. *New Phytologist* **208**(1): 96-101.
